# Drug-induced gastric motility disorders: A disproportionality analysis from the FAERS and CVARD databases

**DOI:** 10.1371/journal.pone.0351731

**Published:** 2026-06-12

**Authors:** Zhiheng Qian, Ni Jiang

**Affiliations:** Jinhua Central Hospital, Jinhua, China; Universitas Airlangga Fakultas Kedokteran, INDONESIA

## Abstract

**Background:**

Delayed gastric emptying and gastroesophageal reflux represent critical yet frequently underrecognized complications in hospitalized patients, particularly in the context of polypharmacy. While multiple medication classes have been implicated in disrupting gastrointestinal motility, the comprehensive risk spectrum of individual drugs remains poorly characterized. This study aimed to conduct a comprehensive disproportionality analysis to identify drugs associated with delayed gastric emptying and reflux using large-scale pharmacovigilance data.

**Methods:**

We analyzed adverse event reports from the FDA Adverse Event Reporting System (FAERS; 2004–2025; n > 58 million) and validated findings against the Canada Vigilance Adverse Reaction Online Database (CVARD). Disproportionality analysis was performed using Reporting Odds Ratio (ROR), Proportional Reporting Ratio (PRR), and Bayesian Confidence Propagation Neural Network (BCPNN). Weibull time-to-onset analysis was conducted to characterize temporal patterns of adverse event onset.

**Results:**

Among the top 50 drugs screened, 20 demonstrated positive signals across all three algorithms. Glucagon-like peptide-1 (GLP-1) receptor agonists exhibited the strongest associations with gastric motility disorders, with semaglutide showing the highest ROR for impaired gastric emptying (ROR: 80.27; 95% CI: 76.39–84.34), validated in CVARD (ROR: 54.17). Insulin formulations, particularly insulin degludec (ROR: 18.90), bisphosphonates, angiotensin receptor blockers, and trofinetide also demonstrated significant signals. Weibull analysis revealed divergent temporal patterns, ranging from early-onset (trofinetide: median 6.6 days) to late-onset (immunoglobulin G: median 535.1 days).

**Conclusion:**

This study identifies a broad spectrum of drug-associated gastric motility disorders with distinct temporal profiles. These findings provide evidence-based priorities for enhanced pharmacovigilance and inform clinical decision-making to mitigate this preventable cause of morbidity.

## 1 Introduction

Delayed gastric emptying and gastroesophageal reflux represent critical yet frequently underrecognized complications in hospitalized patients, particularly in intensive care settings where polypharmacy is ubiquitous. Recent evidence indicates that up to 60% of ICU patients experience some form of gastrointestinal dysmotility, with those receiving mechanical ventilation being disproportionately affected [1]. The prevalence of feeding intolerance—a clinical manifestation of gastric motility disorders characterized by high gastric residual volumes, vomiting, or inability to achieve nutritional targets—ranges from 2% to 75% across ICU settings, with a median prevalence of approximately 38% [2]. These disturbances not only compromise nutritional support but may also contribute to gut-derived systemic inflammatory responses and immune dysregulation, thereby exacerbating critical illness and propagating downstream organ dysfunction [3–5]. In surgical populations, postoperative gastric dysmotility has been identified as a significant determinant of prolonged recovery and increased healthcare resource utilization, with enhanced recovery protocols demonstrating reduced delayed gastric emptying rates and shorter hospital stays [6,7]. The clinical significance of these findings is underscored by the observation that patients with delayed gastric emptying frequently require prolonged nutritional support and experience higher rates of postoperative complications.

While the pathophysiology of gastric dysmotility in critical illness is multifactorial involving inflammatory mediators, electrolyte imbalances, and autonomic dysfunction, emerging evidence underscores the pivotal role of pharmacotherapy as a potentially modifiable risk factor [1,8]. Multiple medication classes commonly prescribed in hospitalized settings have been implicated in disrupting gastrointestinal motility through diverse mechanisms ranging from direct smooth muscle inhibition to alterations in enteric nervous system signaling and reduced lower esophageal sphincter pressure [8,9]. Moreover, bedside-detectable reflux events during mechanical ventilation and enteral feeding have been described in recent clinical observations, highlighting the relevance of reflux as a practical ICU problem rather than a purely theoretical concern [10]. However, the comprehensive risk spectrum of individual drugs and their relative propensity to cause delayed gastric emptying and reflux remains poorly characterized. Existing studies have primarily focused on select drug categories or specific patient populations, leaving substantial gaps in our understanding of the broader pharmacological landscape contributing to these adverse events across diverse clinical contexts. This knowledge deficit limits the ability of clinicians to make informed prescribing decisions that balance therapeutic benefits against gastrointestinal risks.

The clinical consequences of drug-induced gastric motility disorders are severe and well-documented. Gastric distention resulting from delayed emptying increases the risk of vomiting and aspiration, leading to aspiration pneumonia with reported 30-day mortality rates of 21%—fivefold higher than that of community-acquired pneumonia [11]. Meta-analytic evidence demonstrates that feeding intolerance is significantly associated with increased mortality (odds ratio 1.44, 95% CI: 1.35–1.53) and prolonged ICU length of stay (mean difference +3.41 days), with higher feeding intolerance days showing a significant dose-response relationship with mortality outcomes [12]. Beyond these immediate complications, delayed gastric emptying may compromise enteral medication absorption, potentially resulting in subtherapeutic drug concentrations and treatment failure—a frequently overlooked yet clinically significant consequence that may further compromise patient outcomes [13]. The economic burden is equally substantial, with gastroparesis-related emergency department visits increasing by 138% over an eight-year period and associated healthcare charges exceeding $592 million annually [14].

Despite these compelling associations, current clinical practice often fails to adequately consider the gastrointestinal side effects of prescribed medications when formulating therapeutic regimens. Gastrointestinal motility disturbances are frequently attributed to the underlying disease process rather than iatrogenic causes, leading to missed opportunities for prevention and intervention [9,15]. The absence of standardized definitions and validated assessment tools for drug-induced gastric dysmotility has further hampered systematic investigation and risk stratification; a recent systematic review identified 89 unique definitions of feeding intolerance across the literature, highlighting the heterogeneity that impedes comparative analyses [2]. Large-scale pharmacovigilance data that could inform evidence-based prescribing decisions and identify high-risk medications at the population level remain scarce, limiting the development of targeted prevention strategies and clinical decision support tools.

To address these critical knowledge gaps, this study leverages the FDA Adverse Event Reporting System (FAERS)—the largest publicly available pharmacovigilance database encompassing millions of adverse event reports from diverse clinical settings worldwide—to conduct a comprehensive disproportionality analysis of drugs associated with delayed gastric emptying and reflux. By systematically quantifying reporting risks across the pharmacological spectrum and identifying the top 20 medications with the strongest disproportionality signals, we aim to establish evidence-based priorities for enhanced monitoring, guide clinicians in making informed prescribing decisions, and ultimately mitigate this preventable cause of morbidity and mortality in hospitalized patients.

## 2 Methods

### 2.1 Study design

This study was designed as a multi-step pharmacovigilance analysis to identify drugs associated with delayed gastric emptying and reflux-related adverse events. First, disproportionality analysis was performed using the FAERS database to detect drug–event pairs with disproportionate reporting and to screen signal-positive drugs potentially associated with increased aspiration risk. Signal detection in FAERS was based on concordant positivity across three algorithms, including the reporting odds ratio (ROR), proportional reporting ratio (PRR), and Bayesian confidence propagation neural network (BCPNN).

**Fig 1:**
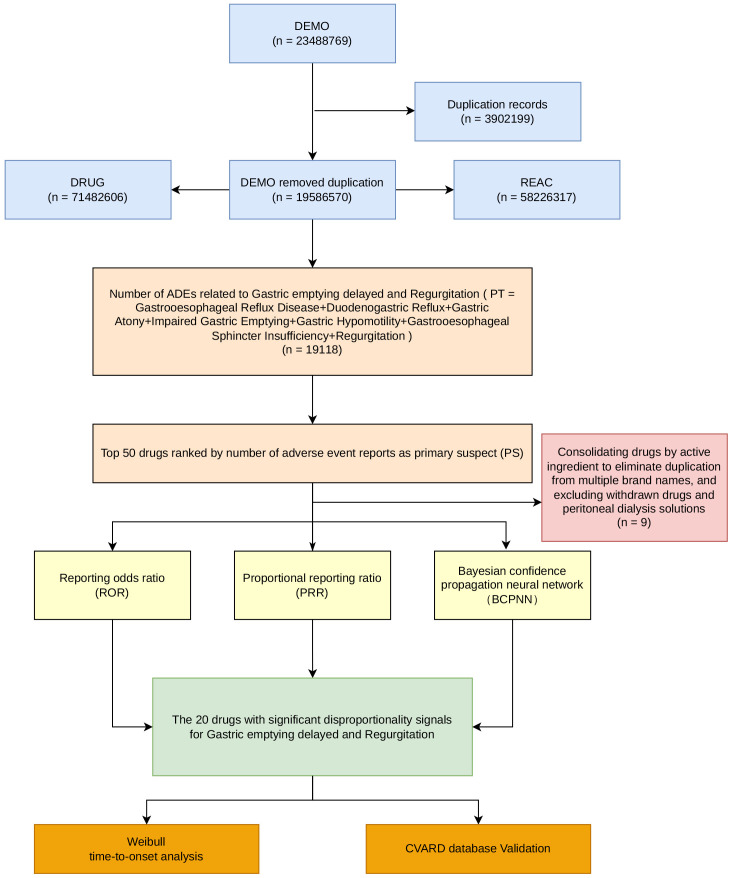
Flowchart of Research Design. DEMO, Patient demographics and administrative table, each record represents a single report in the database; RACE, Reactions table, all adverse events that occurred in patients after medication use were recorded; DRUG, drug table, record all medication information related to the adverse event report; ADEs, Adverse Drug Events; PT, Preferred Term.

Second, the identified signals were externally evaluated using the Canada Vigilance Adverse Reaction Online Database (CVARD). Given the substantial difference in database size between FAERS and CVARD, differential positivity criteria were applied for validation purposes. In FAERS, signals were defined by simultaneous positivity across all three algorithms, whereas in CVARD, signals were evaluated using ROR disproportionality alone, defined as a case count of at least 3 and a lower 95% confidence interval greater than 1. Finally, Weibull time-to-onset analysis was conducted for signal-positive drugs to compare onset patterns and characterize the temporal profiles of these adverse events across different pharmacological agents. The overall study workflow is shown in Fig 1.

### 2.2 Data sources and data cleaning

The data were sourced from the U.S. Food and Drug Administration (FDA) Adverse Event Reporting System (FAERS) database (https://fis.fda.gov/extensions/FPD-QDE-FAERS/FPD-QDE-FAERS.html). This study utilized the raw adverse event report data in ASCII format obtained from the website, covering a total of 87 quarters from the first quarter of 2004 to the third quarter of 2025 (approximately 21.75 years). The MySQL database management system was used to store, manage, and integrate the database, which contains information such as drug-related adverse events, adverse event reporting dates and outcomes, routes of administration, dosages, cumulative dosages, patient gender and age, and reporting countries. Additionally, data from the Canada Vigilance Adverse Reaction Online Database (CVARD, https://www.canada.ca/en/health-canada/services/drugs-health-products/medeffect-canada/adverse-reaction-database/medeffect-canada-caveat-privacy-statement-interpretation-data-search-canada-vigilance-adverse-reaction-online-database.html) were obtained for the period from December 31, 1964, to August 31, 2025, to serve as a validation dataset. The above data were downloaded for the study on January 14, 2026, and the authors were not authorized to identify participant information.

Because the FAERS database spans 87 quarters, substantial variation exists in data structure and reporting formats across different periods. To ensure consistency, all FAERS data were imported, stored, and integrated in MySQL. Using the official documentation for the third quarter of 2025 as the harmonization standard, data from all quarters were standardized through SQL-based processing, including string mapping, format harmonization, and data type conversion, to make the dataset suitable for subsequent statistical analysis. A similar cleaning and integration procedure was applied to the CVARD data. According to the Medical Dictionary for Regulatory Activities (MedDRA), preferred terms (PTs) were standardized, and missing or unknown values were processed during data cleaning. Adverse events were classified according to MedDRA System Organ Classes (SOCs). Records containing spelling errors, unrecognizable or meaningless PTs or drug names, and duplicate entries were identified and removed using SQL queries and data validation procedures.

### 2.3 Definition of variables

The following MedDRA preferred terms were established as primary search terms for adverse reactions associated with delayed gastric emptying and reflux: Gastrooesophageal Reflux Disease, Duodenogastric Reflux, Gastric Atony, Impaired Gastric Emptying, Gastric Hypomotility, Gastrooesophageal Sphincter Insufficiency, Regurgitation.

### 2.4 Statistical analysis

Data processing and statistical analysis were performed using Microsoft Excel and R (4.4.2) software, with the data.table and tidyverse packages for data management and processing, ggplot2 for visualization, and flexsurv for Weibull time-to-onset analysis. Signal mining was based on the four-fold table (S1 File), and calculations were carried out using the reporting odds ratio (ROR), the proportional reporting ratio (PRR), and the Bayesian confidence propagation neural network (BCPNN). For each drug–preferred term pair, disproportionality analysis was performed using a standard 2 × 2 contingency table, in which the reference framework consisted of reports involving the target drug with other adverse events and reports involving other drugs with and without the adverse event of interest. If a result met the prespecified requirements of all three algorithms simultaneously, it was defined as one risk signal. Briefly, ROR required the lower bound of the 95% confidence interval (CI) to exceed 1; PRR required PRR ≥ 2, χ² ≥ 4, and at least 3 cases; and BCPNN required the lower bound of the 95% CI of the information component (IC025) to exceed 0. The calculation formulas and detailed judgment criteria are shown in S2 File. Signal strength was determined by the ROR value, with larger ROR values indicating stronger disproportionality among signal-positive drug–event pairs. Although signal detection was based on concordance across ROR, PRR, and BCPNN, ROR was used in the Results primarily as an intuitive measure to present the relative magnitude of these already signal-positive drug–event pairs.

## 3 Results

### 3.1 Drugs associated with delayed gastric emptying and reflux

**Fig 2:**
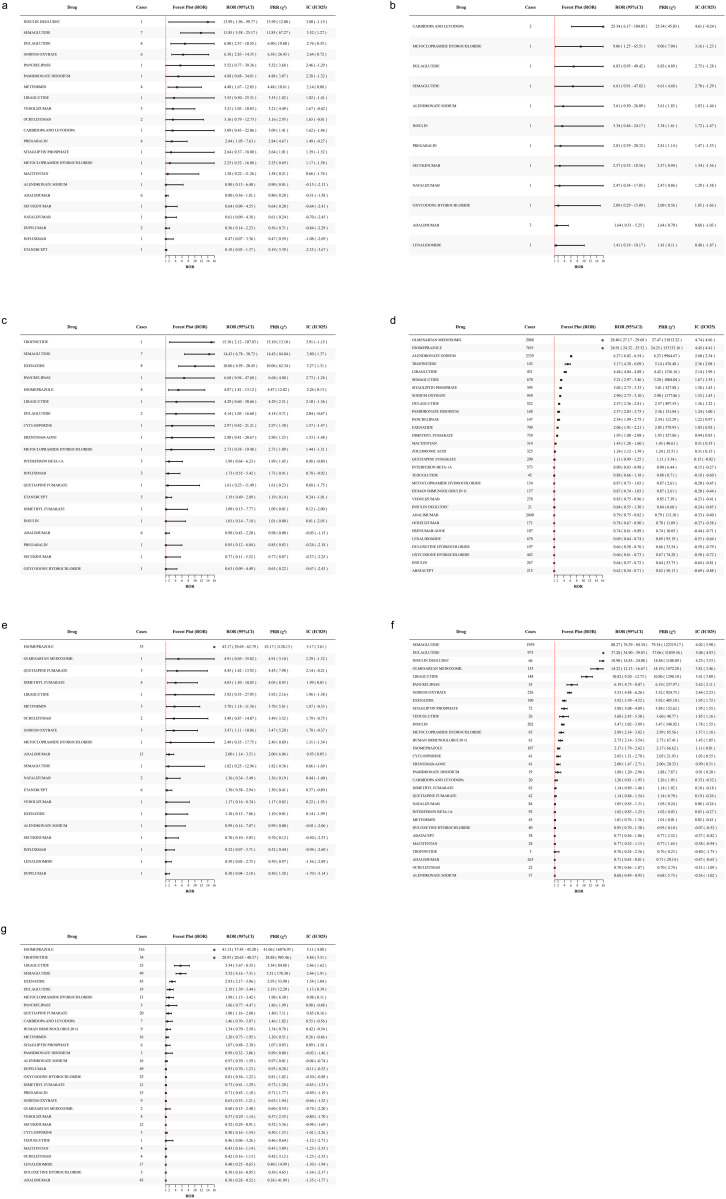
Forest plots of disproportionality estimates for drug–event pairs. Panels show (a) duodenogastric reflux, (b) gastric atony, (c) gastric hypomotility, (d) gastrooesophageal reflux disease, (e) gastrooesophageal sphincter insufficiency, (f) impaired gastric emptying, and (g) regurgitation. Open circles indicate reporting odds ratio (ROR) point estimates, and horizontal lines indicate 95% confidence intervals (CIs). PRR, proportional reporting ratio; IC, information component in the Bayesian confidence propagation neural network; IC025, the lower limit of the 95% CI of the IC. For some drug–event pairs with relatively large case counts, the 95% CIs were extremely narrow and may visually overlap with the point estimates. For some pairs with very large ROR values or wide confidence intervals, the estimates extend beyond the plotted x-axis range and are therefore truncated at the plot boundary for visualization.

Among the top 50 drugs associated with gastrointestinal reflux and delayed gastric emptying, disproportionality analysis identified 20 drugs demonstrating positive signals that simultaneously met the thresholds of Reporting Odds Ratio (ROR), Proportional Reporting Ratio (PRR), and Bayesian Confidence Propagation Neural Network (BCPNN). Signal strengths for each drug, categorized by MedDRA Preferred Term (PT), are illustrated in Fig 2.

As shown in Fig 2, the 20 signal-positive drugs, listed in descending order according to the highest ROR observed across all signal-positive preferred terms for each drug, were: Semaglutide, Esomeprazole, Dulaglutide, Trofinetide, Olmesartan medoxomil, Insulin degludec, Liraglutide, Exenatide, Sodium oxybate, Alendronate sodium, Pancrelipase, Metformin, Dimethyl fumarate, Sitagliptin phosphate, Teduglutide, Insulin, Metoclopramide hydrochloride, Human immunoglobulin G, Pamidronate disodium, and Cyclosporine. A summary table is provided in S3 File.

### 3.2 Baseline characteristics of delayed gastric emptying and reflux

In the 20 signal-positive drugs analyzed, a total of 22,601 adverse event reports were identified, among which 14,011 were associated with female patients (62.0%) and 4,802 with male patients (21.2%). The proportion of female patients significantly exceeded that of males, although gender information was missing in 16.8% of reports. Regarding age distribution, 40.4% of reports lacked age data. Among the remaining reports with available information, the largest proportion fell within the 45–59 years age group (23.4%), followed by the 60–74 years category (20.4%). The elderly population (≥60 years) collectively accounted for 25.5% of reports with documented age data.

Concerning the time interval from drug initiation to event onset, 67.99% of reports were missing duration information. Among the documented cases, the majority of events occurred within 0–30 days (8.18% of the total), while events occurring beyond 360 days represented 6.06% of the total. With respect to clinical outcomes, the majority were classified as “other serious” (OT, 46.95%), followed by hospitalization (HO, 33.32%). Serious outcomes including death (DE, 2.85%), life-threatening events (LT, 3.26%), and disability (DS, 6.79%) collectively comprised 12.9% of reports. Detailed baseline characteristics are presented in Table 1.

**Table 1:** Baseline patient data in signal-positive adverse drug event reports.

Dimension	Classification	Number	Percent(%)
Sex	F	14011	62.0
	M	4802	21.2
	Missing	3788	16.8
Age (Years)	90+	48	0.2
	75-89	1103	4.9
	60-74	4602	20.4
	45-59	5289	23.4
	18-44	2239	9.9
	0-17	180	0.8
	Missing	9140	40.4
Duration	0-30 d	1947	8.18
	31-60 d	451	1.89
	61-90 d	267	1.12
	91-120 d	172	0.72
	121-150 d	144	0.60
	151-180 d	125	0.52
	181-360 d	572	2.40
	360 d+	1443	6.06
	Missing	16183	67.99
Outcome	OT	8851	46.95
	HO	6281	33.32
	DS	1281	6.79
	LT	615	3.26
	DE	537	2.85
	RI	57	0.30
	CA	57	0.30

Duration: Time from drug initiation to adverse event onset.

Outcome codes: OT (Other), HO (Hospitalization), DS (Disability), LT (Life-threatening), DE (Death), RI (Required Intervention), CA (Congenital Anomaly).

### 3.3 Signal distribution across drug–event pairs

**Fig 3:**
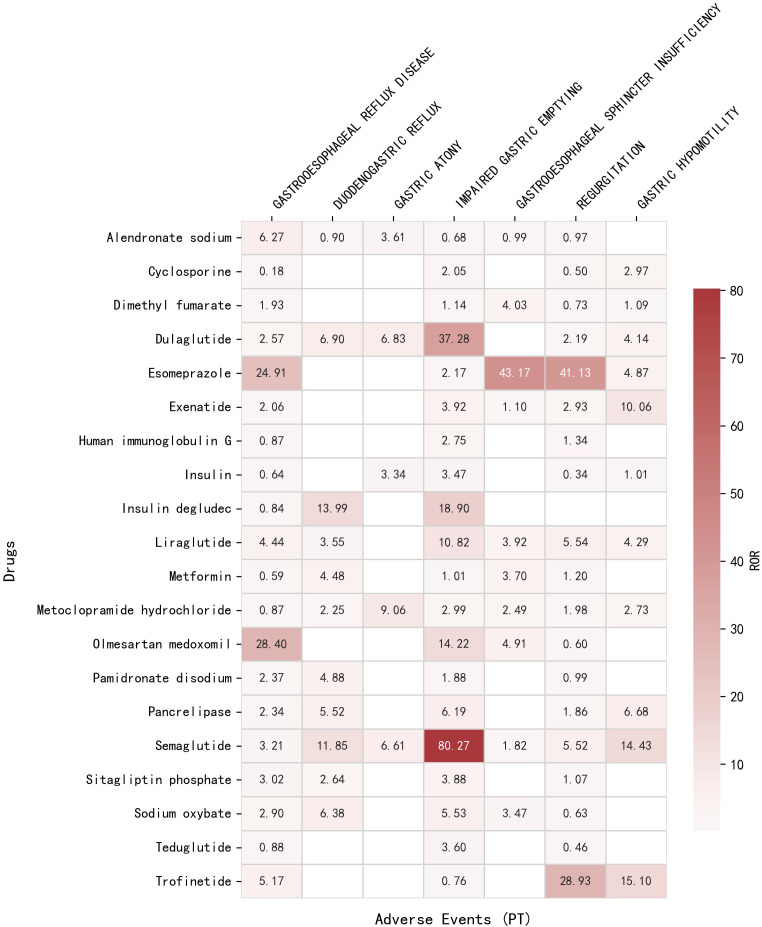
Heatmap of reporting odds ratio (ROR) values for 20 signal-positive drugs across seven preferred terms (PTs). Rows represent drugs and columns represent PTs. Cell colors indicate the magnitude of the ROR, and the numeric annotations within cells show the corresponding raw ROR values. Blank cells indicate that no corresponding drug–PT entry was retained in the input dataset for that drug, rather than a separate color-based signal threshold. ROR = 1 represents the null reference for disproportionality. This figure is intended to visualize the relative pattern of ROR values across drug–event pairs; formal signal positivity was defined according to the prespecified concordant criteria described in the Methods section.

A heatmap of Reporting Odds Ratios (RORs) was generated to visualize the strength of association between 20 signal-positive drugs and seven gastrointestinal adverse event preferred terms (PTs), including gastroesophageal reflux disease, duodenogastric reflux, gastric atony, impaired gastric emptying, gastroesophageal sphincter insufficiency, regurgitation, and gastric hypomotility (Fig 3).

The analysis revealed marked heterogeneity in signal distribution across drug classes. Notably, glucagon-like peptide-1 (GLP-1) receptor agonists exhibited pronounced signals for delayed gastric emptying and motility disorders. Specifically, semaglutide demonstrated the strongest association with impaired gastric emptying (ROR: 80.27, 95% CI: 76.39–84.34), followed by dulaglutide (ROR: 37.28, 95% CI: 34.90–39.83) and liraglutide (ROR: 10.82, 95% CI: 9.20–12.73). Additionally, exenatide and semaglutide showed elevated RORs for gastric hypomotility (10.06, 95% CI: 4.95–20.45; and 14.43, 95% CI: 6.78–30.73, respectively), suggesting a class-level safety signal related to gastrointestinal transit inhibition.

Conversely, esomeprazole displayed disproportionate reporting for gastroesophageal sphincter insufficiency (ROR: 43.17, 95% CI: 29.69–62.79) and regurgitation (ROR: 41.13, 95% CI: 37.43–45.20), which may reflect prescription channeling bias given its therapeutic indication for acid-related disorders. Similarly, trofinetide exhibited high RORs for regurgitation (ROR: 28.93, 95% CI: 20.63–40.57) and gastric hypomotility (ROR: 15.10, 95% CI: 2.12–107.83). Among other agents, olmesartan medoxomil showed a strong signal for gastroesophageal reflux disease (ROR: 28.40, 95% CI: 27.17–29.68), while insulin degludec was associated with impaired gastric emptying (ROR: 18.90, 95% CI: 14.83–24.08). Metoclopramide hydrochloride, a prokinetic agent, demonstrated unexpectedly high RORs for gastric atony (ROR: 9.06, 95% CI: 1.25–65.51) and impaired gastric emptying (ROR: 2.99, 95% CI: 2.34–3.82).

### 3.4 Disproportionality analysis of the CVARD database for validation purposes

**Fig 4:**
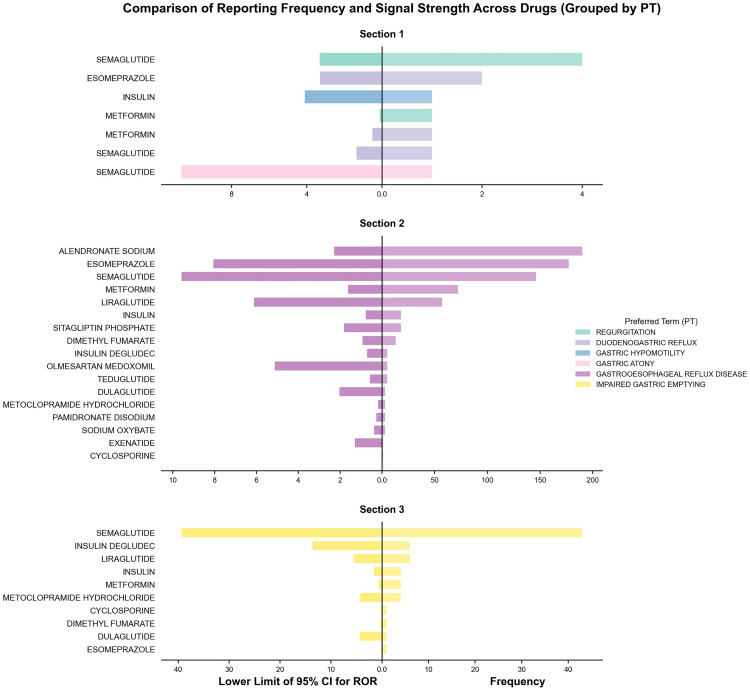
Drug frequency and ROR signal value in CVARD database. In the CVARD database, with the exception of human immunoglobulin G, pancrelipase, and trofinetide which were not identified, the signal strengths of the remaining drugs are shown in the figure, including the reporting odds ratios (RORs) on the left and the reporting frequencies on the right.

Of the 20 signal-positive drugs, human immunoglobulin G, pancrelipase, and trofinetide were not found in the CVARD database. The results of the Disproportionality Analysis for the remaining drugs are presented in Fig 4.

#### 3.4.1 Consistent signals across databases.

Thirteen drug-Preferred Term (PT) combinations demonstrated robust cross-database validation. Glucagon-like peptide-1 (GLP-1) receptor agonists exhibited the most pronounced and consistent associations with gastric motility disorders. Semaglutide demonstrated the strongest signal for impaired gastric emptying (FAERS ROR: 80.27, 95% CI: 76.39–84.34; CVARD ROR: 54.17, 95% CI: 39.34–74.59), with both databases additionally identifying significant signals for gastrooesophageal reflux disease (GERD) and regurgitation. Similarly, liraglutide showed consistent positive signals for impaired gastric emptying (FAERS ROR: 10.82, 95% CI: 9.20–12.73; CVARD ROR: 12.37, 95% CI: 5.52–27.73) and GERD (FAERS ROR: 4.44, 95% CI: 4.04–4.88; CVARD ROR: 7.96, 95% CI: 6.12–10.34). Dulaglutide and exenatide exhibited concordant signals for GERD, though exenatide in CVARD was limited by small case counts (n = 1).

Other consistently validated signals included insulin degludec (impaired gastric emptying: FAERS ROR: 18.90, 95% CI: 14.83–24.08; CVARD ROR: 30.54, 95% CI: 13.61–68.51) and insulin (FAERS ROR: 3.47, 95% CI: 3.02–3.99; CVARD ROR: 4.08, 95% CI: 1.52–10.94), suggesting a class-level effect of insulin formulations on gastric motility. Alendronate sodium, esomeprazole, olmesartan medoxomil, metoclopramide hydrochloride, and sitagliptin phosphate all demonstrated convergent signals for GERD or impaired gastric emptying across both databases, notwithstanding variations in signal magnitude. The table of common positive results from both databases is presented in Table 2.

**Table 2:** Drugs with consistent signals across databases.

Drug	Adverse Reaction Term (PT)	FAERS ROR (95% CI)	CVARD ROR (95% CI)
Semaglutide	Impaired gastric emptying	80.27 (76.39–84.34)	54.17 (39.34–74.59)
	Gastrooesophageal reflux disease	3.21 (2.97–3.46)	11.31 (9.58–13.34)
	Regurgitation	5.52 (4.16–7.31)	8.93 (3.31–24.07)
Liraglutide	Impaired gastric emptying	10.82 (9.20–12.73)	12.37 (5.52–27.73)
	Gastrooesophageal reflux disease	4.44 (4.04–4.88)	7.96 (6.12–10.34)
Dulaglutide	Gastrooesophageal reflux disease	2.57 (2.36–2.81)	6.32 (2.03–19.68)
Exenatide	Gastrooesophageal reflux disease	2.06 (1.91–2.21)	*9.27 (1.29–66.51)
Insulin degludec	Impaired gastric emptying	18.90 (14.83–24.08)	30.54 (13.61–68.51)
Insulin	Impaired gastric emptying	3.47 (3.02–3.99)	4.08 (1.52–10.94)
Alendronate sodium	Gastrooesophageal reflux disease	6.27 (6.02–6.54)	2.63 (2.28–3.04)
Esomeprazole	Gastrooesophageal reflux disease	24.91 (24.32–25.52)	9.37 (8.06–10.89)
Olmesartan medoxomil	Gastrooesophageal reflux disease	28.40 (27.17–29.68)	12.38 (5.12–29.94)
Metoclopramide	Impaired gastric emptying	2.99 (2.34–3.82)	11.35 (4.24–30.44)
Sitagliptin phosphate	Gastrooesophageal reflux disease	3.02 (2.73–3.33)	2.89 (1.82–4.59)

*Although exenatide did not meet the ROR positive threshold (a = 1) in the CVARD database, its ROR value was 9.27, indicating a signal trend consistent with that in FAERS.

#### 3.4.2 Signals attenuated by sample size limitations and data absence in CVARD.

Several drugs exhibited strong three-algorithm positivity in FAERS but could not be validated in CVARD due to data limitations. For drugs present in both databases, insufficient case counts (n < 3) or wide confidence intervals spanning unity precluded signal detection, as observed for dulaglutide (impaired gastric emptying: FAERS ROR: 37.28, 95% CI: 34.90–39.83; CVARD n = 1) and cyclosporine (impaired gastric emptying: FAERS ROR: 2.05, 95% CI: 1.51–2.78; CVARD n = 1). In addition, human immunoglobulin G, pancrelipase, and trofinetide were entirely absent from the CVARD database. Negative and missing drugs in the CVARD database are listed in Table 3.

**Table 3:** Drugs with inconsistent signals across databases.

Drug	PT	FAERS ROR (95% CI)	CVARD Status
Dulaglutide	Impaired gastric emptying	37.28 (34.90–39.83)	ROR = 31.08 (4.36–221.80), a = 1
	Duodenogastric reflux	6.90 (2.57–18.55)	No PT record
	Regurgitation	2.19 (1.39–3.44)	No PT record
Exenatide	Impaired gastric emptying	3.92 (3.39–4.52)	No PT record
	Gastric hypomotility	10.06 (4.95–20.45)	No PT record
	Regurgitation	2.93 (2.17–3.96)	No PT record
Cyclosporine	Impaired gastric emptying	2.05 (1.51–2.78)	a = 1
Human immunoglobulin G	Impaired gastric emptying	2.75 (2.14–3.54)	a = 1
Liraglutide	Regurgitation	5.54 (3.67–8.35)	No PT record
Esomeprazole	Gastrooesophageal sphincter insufficiency	43.17 (29.69–62.79)	No PT record
	Regurgitation	41.13 (37.43–45.20)	No PT record
Pancrelipase	Impaired gastric emptying	6.19 (4.75–8.07)	No PT record
Semaglutide	Duodenogastric reflux	11.85 (5.58–25.17)	a = 1
	Gastric hypomotility	14.43 (6.78–30.73)	a = 1
Sitagliptin phosphate	Impaired gastric emptying	3.88 (3.08–4.89)	No PT record
Sodium oxybate	Gastrooesophageal reflux disease	2.90 (2.73–3.10)	ROR = 1.15 (0.37–3.55), a = 3
	Impaired gastric emptying	5.53 (4.88–6.26)	No PT record
Teduglutide	Impaired gastric emptying	3.60 (2.45–5.30)	ROR = 1.37 (0.57–3.29), a = 5
Trofinetide	Gastrooesophageal reflux disease	5.17 (4.38–6.09)	No drug record
	Regurgitation	28.93 (20.63–40.57)	No drug record

^a^Number of reports containing both the target drug and target adverse reaction reports in disproportionality analysis.

Furthermore, for certain drugs present in CVARD—such as exenatide, liraglutide, and esomeprazole—specific PTs including “gastric hypomotility,” “regurgitation,” and “duodenogastric reflux” exhibited zero event counts (i.e., no associated reports), preventing comparative analysis for these specific drug-PT combinations despite their strong signals in FAERS. Additionally, sodium oxybate and teduglutide showed RORs of 1.15 and 1.37 in CVARD, respectively, but with lower 95% confidence intervals <1, rendering them non-significant under the defined criteria despite three-algorithm positivity in FAERS. These discrepancies underscore the limitations of smaller spontaneous reporting databases, including incomplete drug coverage and reduced statistical power for detecting rare adverse events or specific drug-event combinations.

### 3.5 Weibull time-to-onset analysis of signal-positive drugs

Weibull distribution analysis was performed on the 20 signal-positive drugs to characterize the temporal patterns of adverse event onset following drug initiation. The scale parameter (α) and shape parameter (β) were estimated to classify failure types as early-onset, random, or late-onset, thereby informing mechanistic interpretations and clinical risk management strategies. The analysis results are presented in Table 4, and the Weibull analysis plots for each drug are shown in S4 File.

**Table 4:** Weibull Time-to-Onset Analysis of 20 Signal-Positive Drugs.

Drug	Case (n)	TTO (days)		Weibull distribution		Failure type
Scale parameter	Shape parameter
Median (IQR)	Min-max	α (95% CI)	β (95% CI)
Alendronate sodium	2373	163.3 (31.0-731.0)	1-5913	318.38 (280.92-366.41)	0.55 (0.52-0.58)	Early
Cyclosporine	70	19.7 (12.2-34.2)	1-146	30.11 (13.92-54.19)	0.87 (0.65-1.51)	Random
Dimethyl fumarate	808	16.7 (1.0-72.0)	1-2716	43.46 (31.45-60.27)	0.38 (0.36-0.41)	Early
Dulaglutide	1508	88.8 (6.0-478.0)	1-3318	185.47 (142.95-219.31)	0.5 (0.46-0.53)	Early
Esomeprazole	8049	124.7 (1.0-1443.5)	1-11191	332.46 (236.99-458.84)	0.37 (0.35-0.40)	Early
Exenatide	925	7.6 (1.0-36.0)	1-1521	17.89 (14.11-21.61)	0.43 (0.41-0.44)	Early
Human immunoglobulin G	203	535.1 (241.0-1680.0)	1-6005	927.29 (657.48-1380.18)	0.67 (0.53-0.91)	Early
Insulin	471	255.6 (4.0-2158.5)	1-13506	653.03 (212.06-1600.44)	0.39 (0.32-0.54)	Early
Insulin degludec	88	25.8 (6.5-72.5)	1-946	57.4 (9.40-275.95)	0.46 (0.38-1.44)	Random
Liraglutide	590	9 (1.0-29.8)	1-1100	21.53 (15.46-30.94)	0.42 (0.40-0.46)	Early
Metformin	251	29.4 (2.0-142.0)	1-4102	81.02 (30.16-193.72)	0.36 (0.32-0.45)	Early
Metoclopramide hydrochloride	173	668.3 (163.5-2553.5)	1-6882	1161.05 (768.36-1579.77)	0.66 (0.53-0.84)	Early
Olmesartan medoxomil	2161	211.6 (41.8-903.2)	1-4336	408.23 (365.80-463.73)	0.56 (0.53-0.59)	Early
Pamidronate disodium	177	756.7 (320.5-1988.5)	1-4200	1230.42 (769.82-1910.42)	0.75 (0.50-1.27)	Random
Pancrelipase	204	22.6 (1.0-100.5)	1-788	49.88 (17.40-105.90)	0.46 (0.41-0.61)	Early
Semaglutide	2636	28.8 (1.0-153.5)	1-4016	64.62 (55.64-75.51)	0.45 (0.44-0.47)	Early
Sitagliptin phosphate	460	102.5 (7.5-455.5)	1-4285	212.04 (151.25-281.82)	0.5 (0.45-0.56)	Early
Sodium oxybate	1206	48.9 (1.0-371.0)	1-3515	127.07 (62.97-216.14)	0.38 (0.35-0.43)	Early
Teduglutide	72	197.1 (34.8-956.0)	3-3322	376.42 (180.58-754.28)	0.57 (0.49-0.68)	Early
Trofinetide	176	6.6 (1.0-26.0)	1-192	12.49 (6.44-19.89)	0.57 (0.51-0.70)	Early

Weibull distribution fit for time-to-event data.

Abbreviations: TTO, time-to-onset; IQR, interquartile range; CI, confidence interval; Median, median time to event; β, Weibull shape parameter; α, Weibull scale parameter (days).

Failure Type: Early, Failure risk decreases over time, with high incidence of early events, the 95% confidence interval of the shape parameter (β) does not contain 1, and β < 1; Random, The failure risk is constant, while events occur randomly, The 95% confidence interval of the shape parameter (β) contains 1.

The analysis revealed distinct temporal profiles. Glucagon-like peptide-1 (GLP-1) receptor agonists generally demonstrated early-onset patterns. Exenatide exhibited the shortest median time-to-onset (TTO) at 7.6 days (IQR: 1.0–36.0), followed by liraglutide (median TTO: 9.0 days, IQR: 1.0–29.8) and semaglutide (median TTO: 28.8 days, IQR: 1.0–153.5). The short median TTOs for exenatide and liraglutide, together with shape parameter values below 1 (β = 0.43 and 0.42, respectively), suggest a high initial risk that decreases over time, consistent with acute gastrointestinal adaptation mechanisms. Notably, trofinetide demonstrated the most rapid onset among all drugs (median TTO: 6.6 days, IQR: 1.0–26.0; β = 0.57), also supporting an early-onset pattern and suggesting immediate pharmacological effects on gastric motility. Dimethyl fumarate and pancrelipase likewise exhibited relatively short median TTO values of 16.7 days (IQR: 1.0–72.0) and 22.6 days (IQR: 1.0–100.5), respectively, indicating prompt gastrointestinal effects following treatment initiation. In contrast, dulaglutide showed a longer median TTO of 88.8 days (IQR: 6.0–478.0). Although its shape parameter remained consistent with an early-onset pattern (β = 0.50), its larger scale parameter (α = 185.47) suggested a more prolonged temporal profile than that of other GLP-1 receptor agonists.

In addition to these early-onset drugs, several agents demonstrated notably prolonged median TTO values. Human immunoglobulin G showed a markedly prolonged median TTO of 535.1 days (IQR: 241.0–1680.0) with a large scale parameter (α = 927.29), suggesting a prolonged temporal profile that may reflect either true delayed toxicity or confounding by indication in chronic immunological conditions requiring long-term therapy. Metoclopramide hydrochloride displayed a median TTO of 668.3 days (IQR: 163.5–2553.5) with the largest scale parameter (α = 1161.05). This extended latency likely reflects indication bias rather than de novo pharmacological toxicity, given the drug’s therapeutic indication for gastroparesis, where treatment duration correlates with underlying disease chronicity. Alendronate sodium also demonstrated a prolonged median TTO of 163.3 days (IQR: 31.0–731.0) with a relatively large scale parameter (α = 318.38), consistent with a more extended temporal profile that may reflect cumulative mucosal damage mechanisms. Other drugs showed prolonged observed latencies or random-onset patterns. Pamidronate disodium had the longest median TTO among the analyzed drugs, at 756.7 days (IQR: 320.5–1988.5), and was classified as random-onset because the 95% confidence interval of the shape parameter β included 1 [β = 0.75 (95% CI: 0.50–1.27)], suggesting event timing relatively independent of exposure duration. Olmesartan medoxomil exhibited a median TTO of 211.6 days (IQR: 41.8–903.2) with a relatively large scale parameter (α = 408.23), indicating prolonged observed latency rather than a true delayed-onset hazard pattern.

Insulin formulations showed heterogeneous temporal profiles. Regular insulin was classified as early-onset based on a shape parameter below 1 (β = 0.39), although its median TTO was relatively long at 255.6 days (IQR: 4.0–2158.5), with a large scale parameter (α = 653.03), indicating a broad temporal distribution. In contrast, insulin degludec was classified as random-onset because the 95% confidence interval of the shape parameter included 1 [β = 0.46 (95% CI: 0.38–1.44)], with a shorter median TTO of 25.8 days (IQR: 6.5–72.5) and a smaller scale parameter (α = 57.4). This discrepancy may reflect differences in pharmacokinetic profiles—degludec’s ultra-long duration enabling steadier exposure conditions versus the more fluctuating exposure profile of regular insulin—as well as differences in patient characteristics, including glycemic control status and autonomic neuropathy severity. Sodium oxybate was classified as early-onset, with a median TTO of 48.9 days (IQR: 1.0–371.0) and a shape parameter below 1 (β = 0.38), consistent with a declining hazard over time. Teduglutide, a glucagon-like peptide-2 analog, showed a median TTO of 197.1 days (IQR: 34.8–956.0) with a relatively large scale parameter (α = 376.42), suggesting a more prolonged temporal profile that may be related to intestinal adaptation mechanisms.

## 4 Discussion

### 4.1 Principal findings and pharmacological implications

This large-scale pharmacovigilance analysis of over 58 million adverse event reports represents, to our knowledge, the most comprehensive signal detection study characterizing the spectrum of drug-associated gastric motility disorders. Our systematic disproportionality analysis, validated through cross-database comparison with the Canada Vigilance Adverse Reaction Online Database (CVARD), has identified distinct patterns of gastrointestinal adverse events across multiple therapeutic classes, with particularly pronounced signals observed for glucagon-like peptide-1 receptor agonists (GLP-1RAs), insulin formulations, and several other agents. For clarity and brevity, the discussion of signal strength in this study is presented primarily using ROR values, whereas formal signal detection was based on concordance across ROR, PRR, and BCPNN.

### 4.2 GLP-1 receptor agonists: A class effect on gastric motility

The most striking finding of our analysis is the remarkably strong and consistent association between GLP-1RAs and impaired gastric emptying. Semaglutide demonstrated the highest reporting odds ratio (ROR: 80.27) for impaired gastric emptying among all drugs analyzed, followed by dulaglutide (ROR: 37.28) and liraglutide (ROR: 10.82). This represents one of the strongest pharmacovigilance signals ever reported for a gastrointestinal adverse event.

The mechanistic basis for this observation is well-established in the literature. GLP-1 receptors are expressed throughout the gastrointestinal tract, and GLP-1RA exposure slows gastric emptying via coordinated effects on gastric accommodation, pyloric function, and antral motility [16–18]. This “therapeutic side effect” is pharmacologically related to the glucose-lowering mechanism of GLP-1RAs, as delayed gastric emptying reduces postprandial glucose excursions [19]. However, our findings reveal a critical clinical paradox: the very mechanism that contributes to the therapeutic efficacy of GLP-1RAs—delayed gastric emptying—may also be responsible for significant adverse effects when excessive. In STEP trial, once-weekly semaglutide 2.4 mg produced a mean body-weight change of −14.9% at week 68 versus −2.4% with placebo, alongside improvements in cardiometabolic risk factors [20,21]. Yet, our data suggest that a substantial proportion of patients experience clinically significant gastric motility impairment, with semaglutide showing the strongest signal across all algorithms (ROR, PRR, and BCPNN).

The temporal patterns observed in our Weibull analysis provide additional mechanistic insights. Exenatide exhibited the shortest median time-to-onset (median TTO: 7.6 days, IQR: 1.0–36.0), followed by liraglutide (median TTO: 9.0 days, IQR: 1.0–29.8) and semaglutide (median TTO: 28.8 days, IQR: 1.0–153.5). This early-onset pattern suggests a predominantly pharmacodynamic effect rather than cumulative toxicity, consistent with evidence that GLP-1R signaling can rapidly modulate gastric emptying and upper-GI motor function through neurohormonal pathways [22,23]. The cross-database validation strengthens the credibility of our findings. Thirteen drug-PT combinations demonstrated robust consistency between FAERS and CVARD, with GLP-1RAs showing the highest concordance. Semaglutide’s signal for impaired gastric emptying was validated with an ROR of 54.17 in CVARD (compared to 80.27 in FAERS), representing a remarkably consistent signal across independent reporting systems. This external validation is particularly important given concerns about reporting bias in spontaneous reporting databases.

### 4.3 Insulin formulations: An underrecognized class effect

Our analysis identified a consistent class-level effect of insulin formulations on gastric motility. Insulin degludec demonstrated a particularly strong signal (ROR: 18.90 in FAERS; 30.54 in CVARD), while regular insulin showed a more modest but significant association (ROR: 3.47 in FAERS; 4.08 in CVARD). This finding raises important questions about the relationship between insulin therapy and diabetic gastroparesis. The causal interpretation of this association is complex. Diabetic gastroparesis is a well-recognized complication of long-standing diabetes and is commonly linked to autonomic and enteric neuropathy with impaired gastric motility. Therefore, baseline risk and disease duration are important sources of confounding when interpreting insulin–gastroparesis signals [24]. However, our temporal analysis revealed distinct patterns: insulin degludec was classified as random-onset because the 95% confidence interval of the shape parameter β included 1 [β = 0.46 (95% CI: 0.38–1.44)], whereas regular insulin showed early-onset characteristics with a shape parameter below 1 (β = 0.39). This discrepancy may reflect differences in pharmacokinetic profiles—degludec’s ultra-long duration enabling relatively stable exposure at steady state versus regular insulin’s more time-varying exposure—or distinct patient population characteristics including glycemic control status and autonomic neuropathy severity.

The observation that insulin degludec shows a stronger signal than regular insulin is intriguing and warrants further investigation. One possible explanation relates to the pharmacokinetic stability of degludec, which forms a subcutaneous multi-hexamer depot and provides ultra-long, relatively peakless action with reduced peak–trough fluctuations [25,26]. Alternatively, the finding may reflect confounding by indication, as patients prescribed degludec may have more advanced diabetes with greater baseline risk of gastroparesis [24].

### 4.4 Other therapeutic classes: Diverse mechanisms and clinical implications

Beyond GLP-1RAs and insulin, our analysis identified significant signals for several other drug classes, each with distinct mechanistic implications.

**Bisphosphonates:** Alendronate sodium demonstrated a significant association with gastroesophageal reflux disease (GERD; ROR: 6.27 in FAERS; 2.63 in CVARD). This finding is consistent with the known esophageal toxicity of oral bisphosphonates, which can cause mucosal irritation and ulceration [27]. The prolonged median TTO of 163.3 days (IQR: 31.0–731.0) and relatively large scale parameter (α = 318.38) suggest cumulative mucosal damage rather than acute pharmacological effects. Our findings align with previous reports of bisphosphonate-associated esophageal complications and underscore the importance of proper administration instructions (upright posture, adequate water intake, and post-dose fasting) to minimize esophageal exposure [28].

**Angiotensin receptor blockers:** Olmesartan medoxomil exhibited a surprisingly strong signal for GERD (ROR: 28.40 in FAERS; 12.38 in CVARD). While ARBs are generally well-tolerated regarding gastrointestinal effects, this association may relate to the drug’s effects on the renin-angiotensin system, which has been implicated in esophageal motility and lower esophageal sphincter function [29,30]. Alternatively, this signal may reflect channeling bias if olmesartan is preferentially prescribed to patients with comorbid conditions that increase GERD risk.

**Prokinetic agents:** The finding that metoclopramide hydrochloride demonstrated high RORs for gastric atony (9.06) and impaired gastric emptying (2.99) appears paradoxical given its therapeutic indication for gastroparesis. However, this likely represents indication bias rather than true pharmacological toxicity—patients prescribed metoclopramide are, by definition, being treated for gastric motility disorders. The exceptionally prolonged median TTO of 668.3 days (IQR: 163.5–2553.5) and highest scale parameter (α = 1161.05) among all drugs analyzed support this interpretation, as treatment duration correlates with underlying disease chronicity rather than drug-induced toxicity [31].

**Trofinetide:** Trofinetide demonstrated the most rapid onset among all drugs analyzed, with a median TTO of 6.6 days (IQR: 1.0–26.0) and a small scale parameter (α = 12.49). Its shape parameter below 1 (β = 0.57) supported an early-onset pattern, and significant signals were observed for regurgitation (ROR: 28.93) and gastric hypomotility (ROR: 15.10). As a novel insulin-like growth factor-1 (IGF-1) analog approved for Rett syndrome, trofinetide’s rapid gastrointestinal effects warrant particular attention in this vulnerable pediatric population, where feeding difficulties and nutritional compromise are already prevalent comorbidities [32]. The immediate pharmacological effects on gastric motility observed in our analysis suggest that clinicians should implement proactive feeding management protocols, including nutritional assessment, swallowing evaluation, and potential gastrostomy consideration prior to treatment initiation in high-risk patients.

### 4.5 Temporal patterns: Mechanistic insights from Weibull analysis

The Weibull time-to-onset analysis represents a unique strength of our study, providing mechanistic insights beyond traditional disproportionality analysis. The classification of drugs into early-onset, random-onset, and late-onset categories has important implications for understanding underlying mechanisms and guiding clinical monitoring [33,34]. Early-onset patterns, defined by a shape parameter β below 1, were observed for most GLP-1RAs, consistent with direct pharmacological effects on gastric motility. Trofinetide demonstrated the most rapid onset among all drugs analyzed, with a median TTO of 6.6 days (IQR: 1.0–26.0) and a small scale parameter (α = 12.49). Its shape parameter below 1 (β = 0.57) further supported an early-onset pattern, suggesting immediate effects on the central nervous system–gut axis. This is particularly relevant for Rett syndrome patients, in whom trofinetide is indicated, as feeding difficulties and gastrointestinal dysfunction are common comorbidities [35].

Some agents showed prolonged observed latency or random-onset patterns rather than a true late-onset hazard pattern. Human immunoglobulin G exhibited a long median TTO of 535.1 days (IQR: 241.0–1680.0) and a large scale parameter (α = 927.29), suggesting a prolonged temporal profile that may reflect either true delayed toxicity or confounding by indication in chronic immunological conditions requiring long-term therapy. Pamidronate disodium showed a random-onset pattern because the 95% confidence interval of the shape parameter β included 1 [β = 0.75 (95% CI: 0.50–1.27)], indicating event timing relatively independent of exposure duration. Its large scale parameter (α = 1230.42) and prolonged median TTO of 756.7 days (IQR: 320.5–1988.5) suggest a broad temporal distribution rather than a clearly increasing hazard over time [34].

### 4.6 Cross-database validation: Strengthening external validity

The comparative analysis between FAERS and CVARD provides important validation of our findings and highlights the challenges of signal detection across different pharmacovigilance databases. Of the 20 signal-positive drugs identified in FAERS, 13 demonstrated concordant signals in CVARD, with GLP-1RAs showing the highest consistency.

The discrepancies observed between databases are informative. Several drugs exhibited strong three-algorithm positivity in FAERS but could not be validated in CVARD due to insufficient case counts (n < 3) or wide confidence intervals. This reflects the inherent limitations of smaller spontaneous reporting databases, including incomplete drug coverage and reduced statistical power for detecting rare adverse events. For example, dulaglutide showed a strong signal for impaired gastric emptying in FAERS (ROR: 37.28) but only a single case in CVARD, precluding meaningful comparative analysis. And three drugs (human immunoglobulin G, pancrelipase, and trofinetide) were entirely absent from CVARD, likely reflecting differences in drug availability and prescribing patterns between the United States and Canada. This observation underscores the importance of multi-database surveillance for comprehensive signal detection, as no single database captures the full spectrum of drug-event combinations.

### 4.7 Clinical implications and risk mitigation strategies

Our findings have several important clinical implications for the management of patients receiving drugs with established gastric motility effects.

**Risk stratification for GLP-1RAs and dose optimization:** For GLP-1RAs, our data support a risk stratification approach. Patients with pre-existing gastroparesis, prior gastric surgery, or significant gastroesophageal reflux should be considered at higher risk for exacerbation of symptoms. The early-onset pattern (median TTO < 30 days for most GLP-1RAs) suggests that close monitoring during the initial treatment period is warranted, with particular attention to symptoms of delayed gastric emptying including early satiety, postprandial fullness, and nausea [36,37]. The dose-dependent nature of GLP-1RA effects on gastric motility suggests that gradual dose titration may improve tolerability [38]. Current labeling for semaglutide and liraglutide recommends dose escalation over several weeks to minimize gastrointestinal adverse events (Wegovy PI + Saxenda PI), a strategy supported by our temporal analysis showing rapid onset of effects.

**Trofinetide and Rett syndrome: Specialized feeding management:** For trofinetide-treated patients with Rett syndrome, the most rapid onset of gastric motility effects (median TTO: 6.6 days) necessitates specialized feeding management protocols. Given that feeding difficulties are very common among patients with Rett syndrome and aspiration pneumonia is a leading cause of mortality, pretreatment nutritional assessment and swallowing evaluation should be standard of care. Early consideration of gastrostomy may be warranted in patients with significant dysphagia or nutritional compromise [35]. Close monitoring during the first two weeks of therapy, with weekly weight assessments and symptom surveillance, can facilitate early intervention for emerging gastroparesis or regurgitation [39].

**Insulin selection in elderly diabetics:** For elderly patients with diabetes, our findings regarding insulin formulations have important implications for therapeutic selection. The stronger signal observed for insulin degludec compared to regular insulin (ROR: 18.90 vs. 3.47 in FAERS) may reflect the pharmacokinetic stability of ultra-long-acting formulations [40], which produce consistent exposure profiles that could exacerbate underlying diabetic gastroparesis. Elderly patients with long-standing diabetes, autonomic neuropathy, or pre-existing gastrointestinal symptoms may benefit from careful insulin selection, potentially favoring formulations with more predictable pharmacokinetic profiles or considering non-insulin alternatives such as GLP-1RAs or SGLT2 inhibitors when clinically appropriate. However, the benefits of improved glycemic control with modern insulin formulations must be balanced against potential gastrointestinal risks, particularly in frail elderly patients with limited physiological reserve.

For patients who experience intolerable gastric motility effects with one agent, switching to an alternative within the same class may be considered, though our data suggest class effects with quantitative rather than qualitative differences between agents. The observation that exenatide (short-acting) and semaglutide (long-acting) both show significant signals, albeit with different magnitudes, suggests that formulation changes may modulate but not eliminate the gastric motility effects.

### 4.8 Limitations and strengths

Our study has several limitations inherent to pharmacovigilance database analyses. First, spontaneous reporting systems are subject to underreporting, selective reporting, and reporting bias [41]. The “stimulated reporting (notoriety bias)”—where increased media attention or regulatory actions stimulate increased reporting—may amplify signals for high-profile drugs such as GLP-1RAs [42]. We attempted to mitigate this by requiring concordance across multiple disproportionality algorithms and validating findings in an independent database.

Second, the absence of a denominator (total number of patients exposed) precludes calculation of true incidence rates. Our disproportionality metrics (ROR, PRR) assess the relative frequency of reporting compared to other drugs, but cannot determine absolute risk [42]. This limitation is particularly relevant for drugs with rapidly expanding use, such as semaglutide for obesity, where the denominator is increasing exponentially.

Third, confounding by indication is a significant concern, particularly for drugs like metoclopramide that are prescribed specifically for gastric motility disorders. Our Weibull temporal pattern analysis may help identify patterns consistent with acute drug effects vs chronic/indication-related reporting [31], as drugs prescribed for gastroparesis would be expected to show prolonged TTO patterns consistent with chronic disease management rather than acute pharmacological effects.

Fourth, the substantial missing data for time-to-onset (67.99% missing) limits the generalizability of our Weibull analysis. While we used complete case analysis, this may introduce selection bias if missingness is related to outcome severity or reporting patterns [43].

Despite these limitations, our study has several important strengths. The large sample size (over 58 million reports in FAERS) provides statistical power to detect rare adverse events. The use of multiple disproportionality algorithms (ROR, PRR, BCPNN) with stringent positivity criteria reduces false-positive signals. Most importantly, the cross-database validation with CVARD strengthens the external validity of our findings and demonstrates reproducibility across independent reporting systems. Nevertheless, the absence of external validation for some FAERS signals in CVARD should be interpreted cautiously rather than as evidence against the original associations. This discrepancy is likely related, at least in part, to the substantially smaller size of CVARD, which limits statistical power for rare drug–event pairs and may result in wider confidence intervals for disproportionality estimates. Cross-database concordance may also be affected by differences in reporting practices, drug utilization patterns, market availability, and case accrual across countries.

### 4.9 Future directions

Our findings identify several priorities for future research across multiple drug classes. First, prospective studies are needed to quantify the absolute risk of gastric motility disorders across the diverse therapeutic classes identified in our analysis—including GLP-1RAs, insulin formulations, bisphosphonates, and ARBs—and to identify patient-level risk factors that predict susceptibility. Pharmacogenomic studies may reveal genetic variants that modulate drug receptor expression or signaling in the gastrointestinal tract, potentially enabling personalized risk assessment.

Second, mechanistic studies should investigate whether the gastric motility effects observed across different drug classes are reversible upon drug discontinuation and whether tolerance develops with continued exposure. The divergent temporal patterns observed—ranging from the ultra-rapid onset of trofinetide (6.6 days) to the delayed patterns of bisphosphonates (163.3 days) and immunoglobulins (535.1 days)—suggest fundamentally different underlying mechanisms that warrant specific investigation.

Third, comparative effectiveness studies should evaluate whether different formulations within drug classes differ in their gastric motility profiles. For insulin, our data suggest quantitative differences between regular insulin and degludec; similar comparisons for GLP-1RAs (daily vs. weekly, injectable vs. oral), bisphosphonates (daily vs. weekly vs. monthly), and other drug classes could inform therapeutic selection.

Fourth, the potential therapeutic implications of drug-induced gastric emptying modulation warrant exploration. While generally considered adverse effects, delayed gastric emptying may have beneficial applications in specific populations—such as patients with dumping syndrome after bariatric surgery (where GLP-1RAs may be therapeutic) or those requiring enhanced nutrient absorption (where prokinetics may be beneficial). Understanding the dose-response relationships for gastric motility effects across drug classes may enable targeted therapeutic applications.

Fifth, the validation of signals in additional international pharmacovigilance databases—beyond FAERS and CVARD—would strengthen the generalizability of our findings. The observation that three drugs (human immunoglobulin G, pancrelipase, and trofinetide) were entirely absent from CVARD highlights the importance of multi-database surveillance for comprehensive signal detection. In this context, EudraVigilance may provide a broader external validation platform for suspected adverse reaction signals, whereas JADER (the Japanese Adverse Drug Event Report database) may offer complementary evidence from an East Asian reporting population and help assess the consistency of these associations across different regional and demographic settings.

## 5 Conclusion

In conclusion, this comprehensive pharmacovigilance analysis reveals a broad spectrum of drug-associated gastric motility disorders spanning multiple therapeutic classes—from the potent GLP-1 receptor agonists and insulin formulations to bisphosphonates, angiotensin receptor blockers, and novel neurological agents like trofinetide. The identification of divergent temporal patterns—ranging from days (trofinetide, exenatide) to years (immunoglobulins, bisphosphonates)—provides critical insights into underlying mechanisms, distinguishing acute pharmacological effects from cumulative toxicity or indication bias.

The clinical implications are clear: as the use of gastric motility-modulating drugs continues to expand across diverse therapeutic areas—from diabetes and obesity management to osteoporosis treatment and rare neurological disorders—vigilant monitoring of gastrointestinal safety profiles will be paramount to optimize therapeutic outcomes. Healthcare providers should be aware of the distinct temporal patterns of gastric motility effects across drug classes and counsel patients accordingly, with particular attention to those with pre-existing gastrointestinal conditions, elderly patients with long-standing diabetes, and vulnerable pediatric populations such as those with Rett syndrome.

Our cross-database validation approach strengthens confidence in these findings and demonstrates the value of international pharmacovigilance collaboration for signal detection. Future research should focus on quantifying absolute risks across the diverse therapeutic classes identified, identifying susceptibility factors, and developing class-specific strategies to mitigate gastrointestinal adverse effects while preserving therapeutic benefits. The integration of temporal analysis with traditional disproportionality metrics, as demonstrated in this study, represents a methodological advance that may enhance the sensitivity and specificity of pharmacovigilance signal detection for drug-induced gastric motility disorders.

## Supporting information

S1 File Four-fold table of disproportionality analysis. a, number of reports containing both the target drug and target adverse reaction reports; b, number of reports containing other adverse reaction reports of the target drug; c, number of reports containing the target adverse reaction reports of other drugs; d, number of reports containing other drugs and other adverse reaction reports; N, the number of reports. (DOCX)

S2 File Three signal detection algorithms. a, number of reports containing both the target drug and target adverse reaction reports; b, number of reports containing other adverse reaction reports of the target drug; c, number of reports containing the target adverse reaction reports of other drugs; d, number of reports containing other drugs and other adverse reaction reports. 95%CI, 95% confidence interval; N, the number of reports; χ2, chi-squared; IC, information component; IC025, the lower limit of 95% CI of the IC. ROR, reporting odds ratio; PRR, proportional reporting ratio; BCPNN, Bayesian confidence propagation neural network.
(DOCX)

S3 File Summary Table of Signal Values for 20 Positive Drugs. (XLSX)

S4 File Weibull analysis plots. Weibull distribution fit for time-to-event data. The histogram (green bars) represents the observed probability density of time to event, with the red curve indicating the fitted Weibull distribution; Abbreviations: IQR, interquartile range; CI, confidence interval; Median, median time to event; β, Weibull shape parameter; α, Weibull scale parameter (days); Failure Type: Early, Failure risk decreases over time, with high incidence of early events, the 95% confidence interval of the shape parameter (β) does not contain 1, and β < 1; Random, The failure risk is constant, while events occur randomly, The 95% confidence interval of the shape parameter (β) contains 1.
(DOCX)
