# Supplementary material for: Drug-induced gastric motility disorders: A disproportionality analysis from the FAERS and CVARD databases
Source: PLoS One. 2026 Jun 12;21(6):e0351731. doi: 10.1371/journal.pone.0351731 (PMC13262828; doi:10.1371/journal.pone.0351731)
Supplement: S1 File — (DOCX) [file pone.0351731.s001.docx]

Supplementary Material 1. Four-fold table of disproportionality analysis.

| **Item** | **Number of target adverse event reports** | **Number of other adverse event reports** | **Total** |
| --- | --- | --- | --- |
| Target drug | a | b | a + b |
| Other drugs | c | d | c + d |
| Total | a + c | b + d | N = a + b + c + d |

a, number of reports containing both the target drug and target adverse reaction reports; b, number of reports containing other adverse reaction reports of the target drug; c, number of reports containing the target adverse reaction reports of other drugs; d, number of reports containing other drugs and other adverse reaction reports; N, the number of reports.
