# Supplementary material for: Drug-induced gastric motility disorders: A disproportionality analysis from the FAERS and CVARD databases
Source: PLoS One. 2026 Jun 12;21(6):e0351731. doi: 10.1371/journal.pone.0351731 (PMC13262828; doi:10.1371/journal.pone.0351731)
Supplement: S2 File — 95%CI, 95% confidence interval; N, the number of reports; χ2, chi-squared; IC, information component; IC025, the lower limit of 95% CI of the IC. ROR, reporting odds ratio; PRR, proportional reporting ratio; BCPNN, Bayesian confidence propagation neural network. (DOCX) [file pone.0351731.s002.docx]

Supplementary Material 2. Three signal detection algorithms

| **Methods** | **Formula** | **Signal identification criteria** |
| --- | --- | --- |
| ROR | ROR$=\frac{(a/c)}{(b/d)}=$ad/bc  95%CI$=e^{In(ROR)\pm1.96}\sqrt{\frac{1}{a}+\frac{1}{b}+\frac{1}{c}+\frac{1}{d}}$ | a>3; A signal is generated if the lower limit of 95%CI of ROR >1 |
| PRR | PRR$=\frac{a(a+b)}{c(c+b)}$  χ^2^$=\frac{{(\vert ab-cd\vert-N/2)}^{2}\times N}{(a+b)(a+c)(c+d)(b+d)}$ | a>3; PRR>2, χ^2^>4 |
| BCPNN | IC=log_2_a(a+b+c+d)/[(a+c)(a+b)]  IC025 = e^ln(IC) − 1.96(1/a + 1/b + 1/c + 1/d)^0.5^ | IC025>0 |

a, number of reports containing both the target drug and target adverse reaction reports; b, number of reports containing other adverse reaction reports of the target drug; c, number of reports containing the target adverse reaction reports of other drugs; d, number of reports containing other drugs and other adverse reaction reports. 95%CI, 95% confidence interval; N, the number of reports; χ^2^, chi-squared; IC, information component; IC025, the lower limit of 95% CI of the IC. ROR, reporting odds ratio; PRR, proportional reporting ratio; BCPNN, Bayesian confidence propagation neural network.
