# Supplementary material for: Drug-induced gastric motility disorders: A disproportionality analysis from the FAERS and CVARD databases
Source: PLoS One. 2026 Jun 12;21(6):e0351731. doi: 10.1371/journal.pone.0351731 (PMC13262828; doi:10.1371/journal.pone.0351731)

Supplementary Material 4. Weibull analysis plots

Weibull distribution fit for time-to-event data. The histogram (green bars) represents the observed probability density of time to event, with the red curve indicating the fitted Weibull distribution.

Abbreviations: IQR, interquartile range; CI, confidence interval; Median, median time to event; β, Weibull shape parameter; α, Weibull scale parameter (days);

Failure Type: Early, Failure risk decreases over time, with high incidence of early events, the 95% confidence interval of the shape parameter (β) does not contain 1, and β < 1; Random, The failure risk is constant, while events occur randomly, The 95% confidence interval of the shape parameter (β) contains 1.

# ALENDRONATE SODIUM
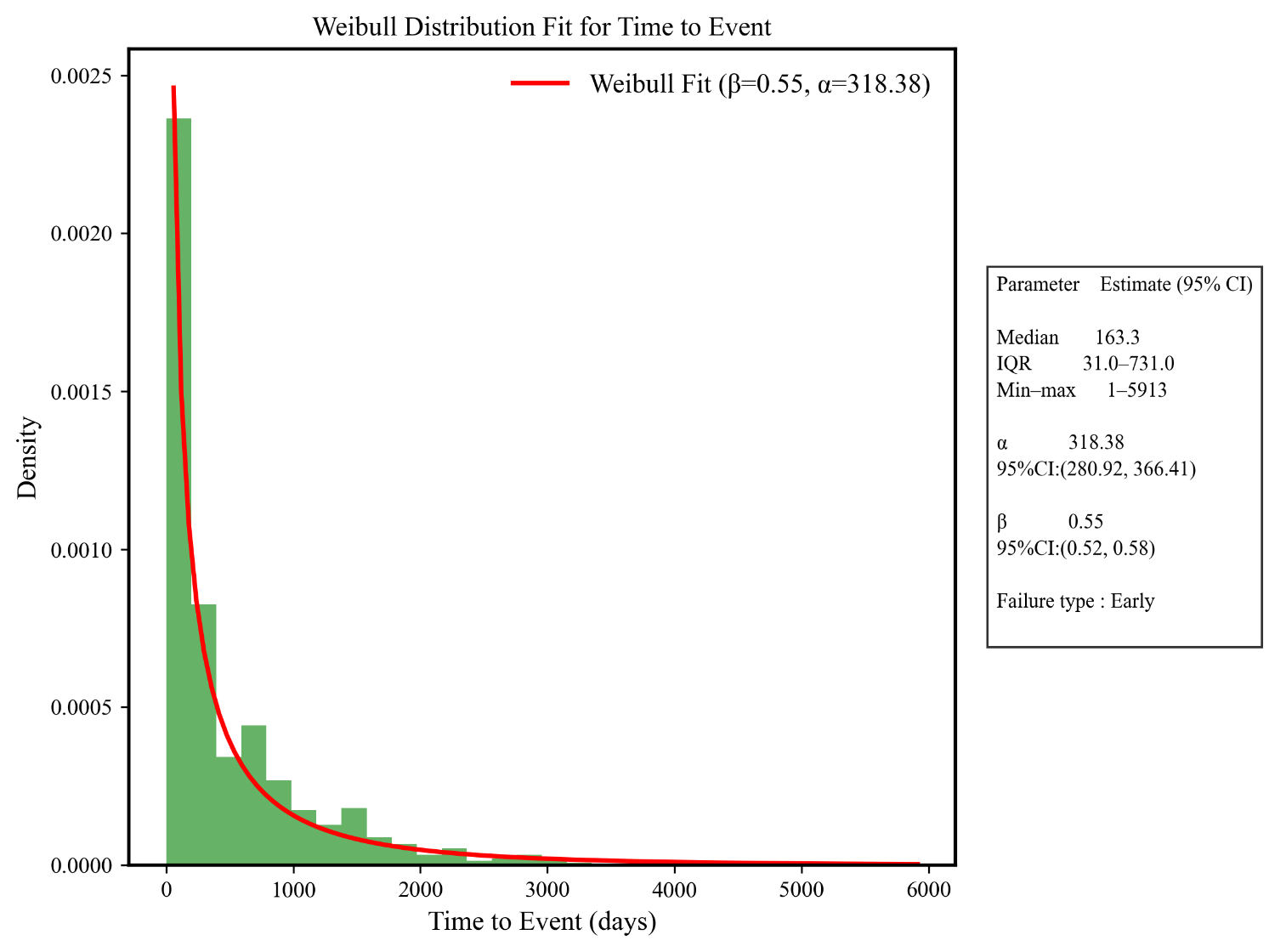


# CYCLOSPORINE


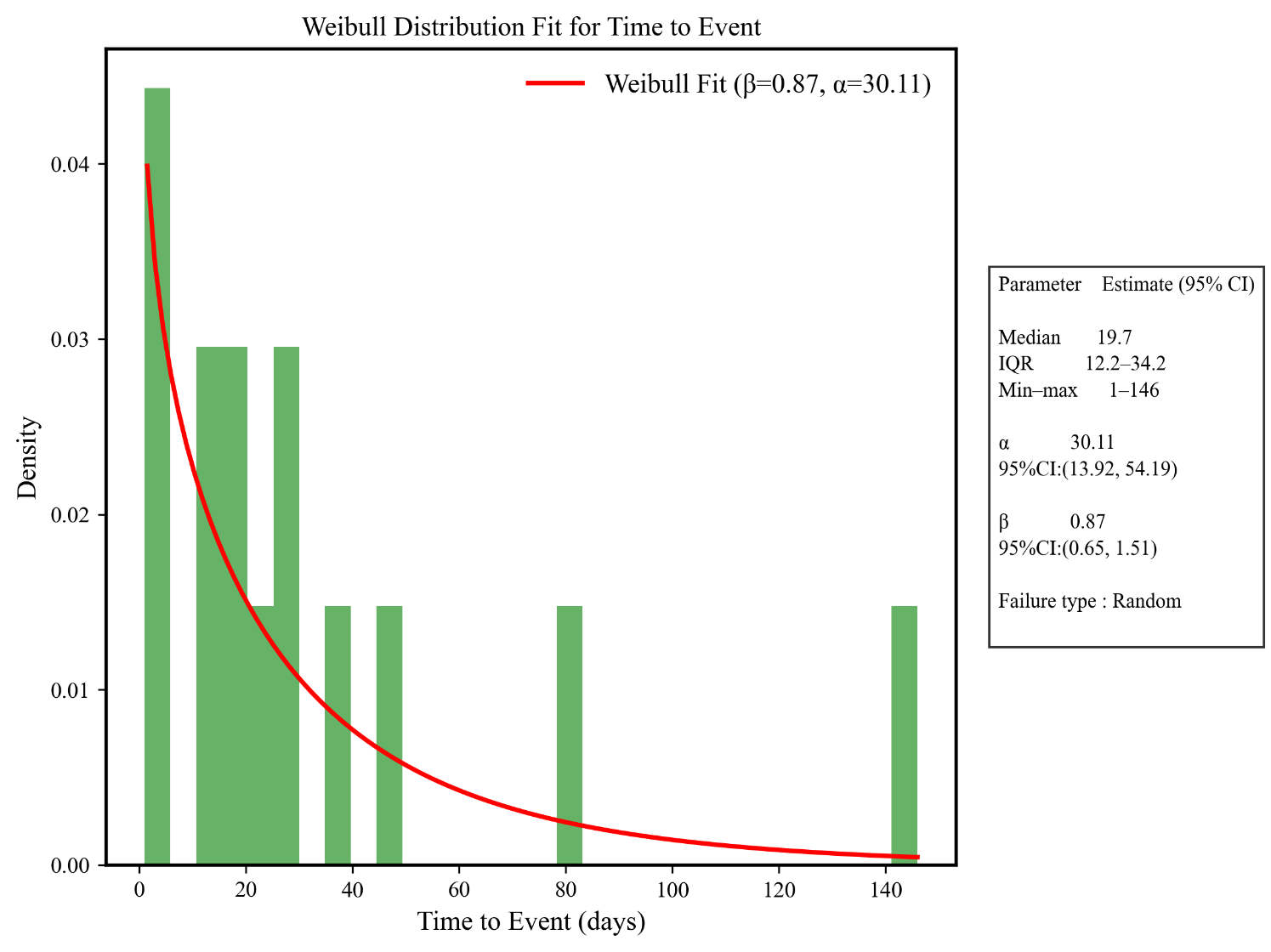


# DIMETHYL FUMARATE


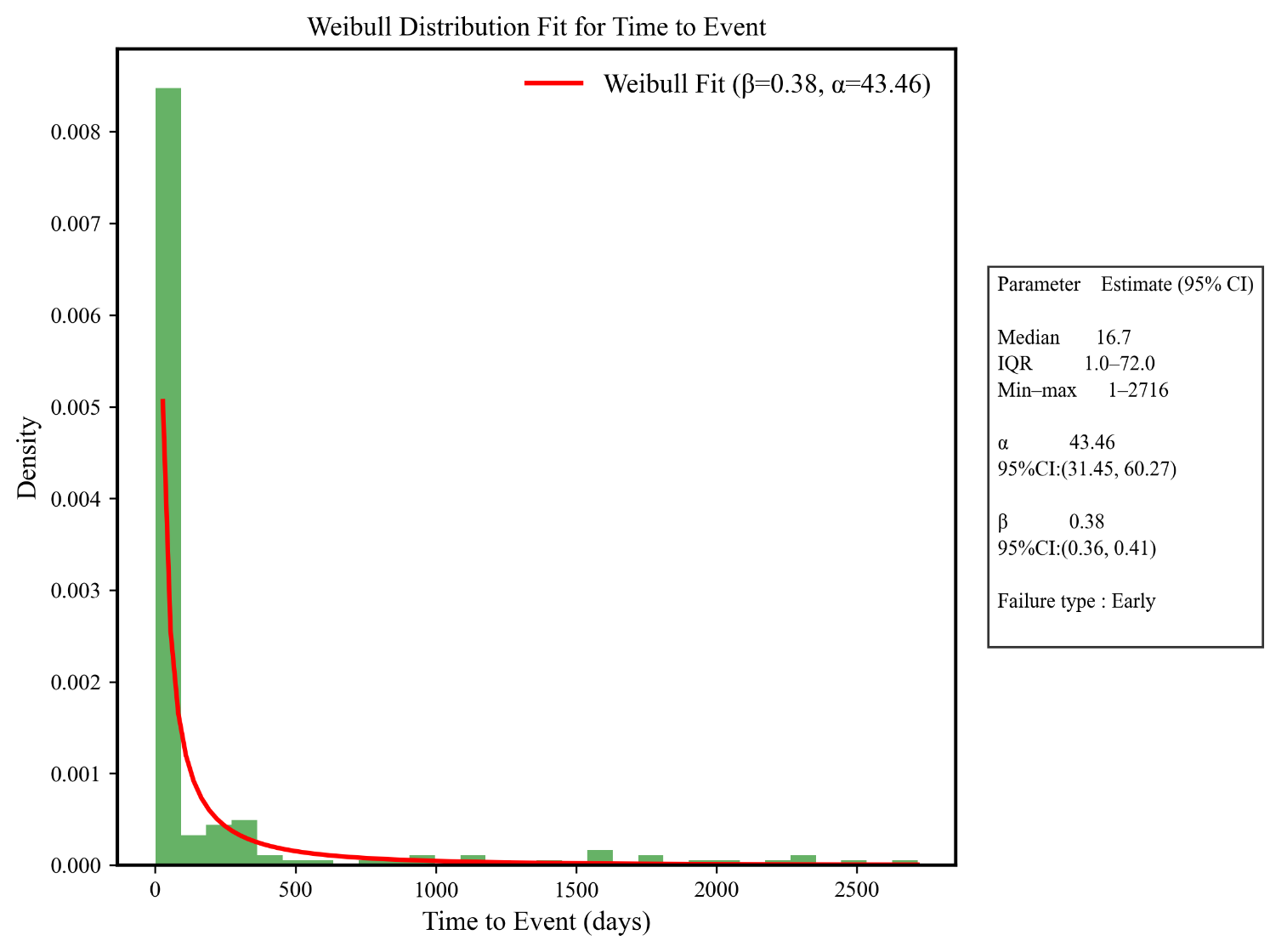


# DULAGLUTIDE


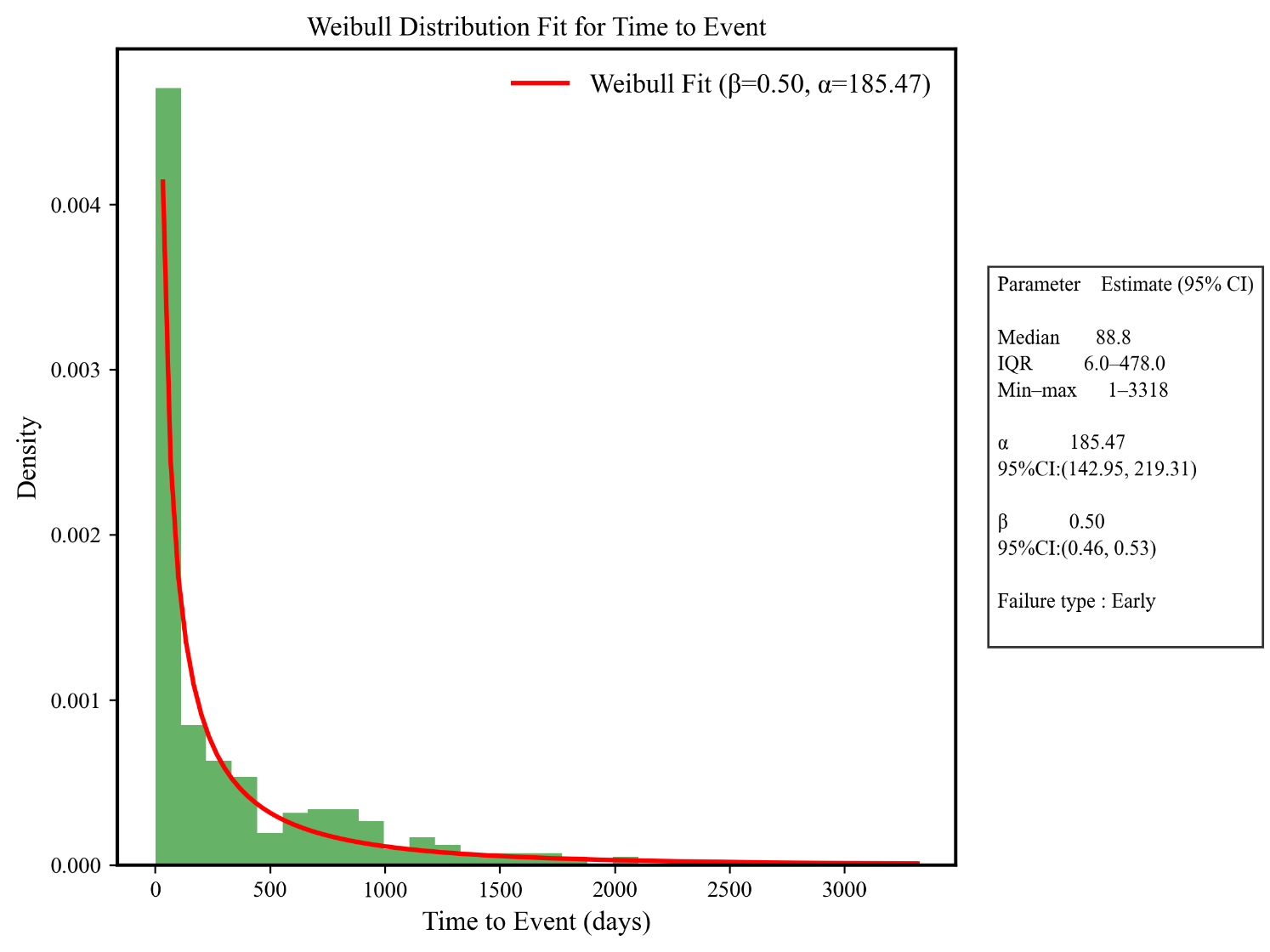


# ESOMEPRAZOLE


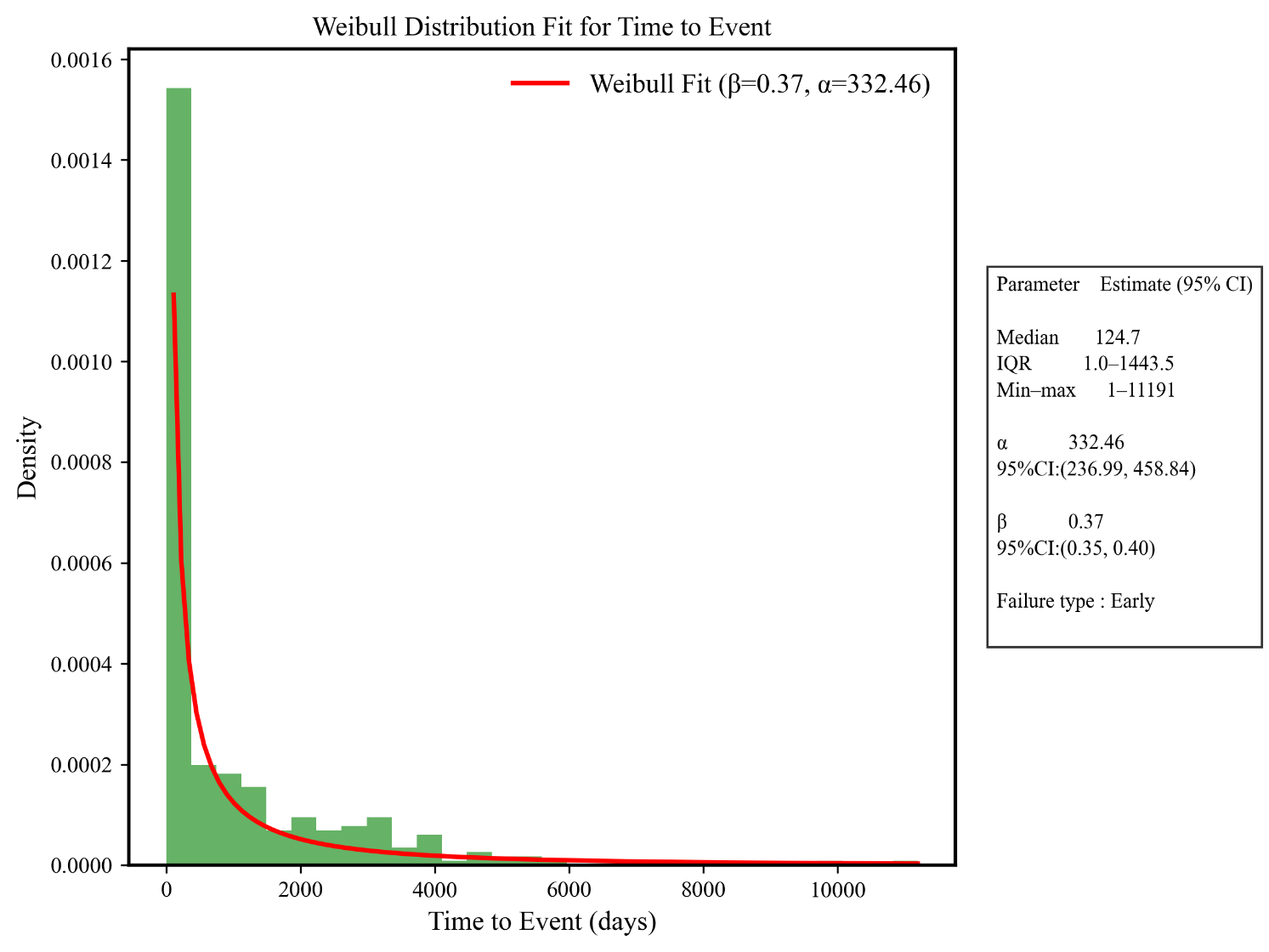


# EXENATIDE


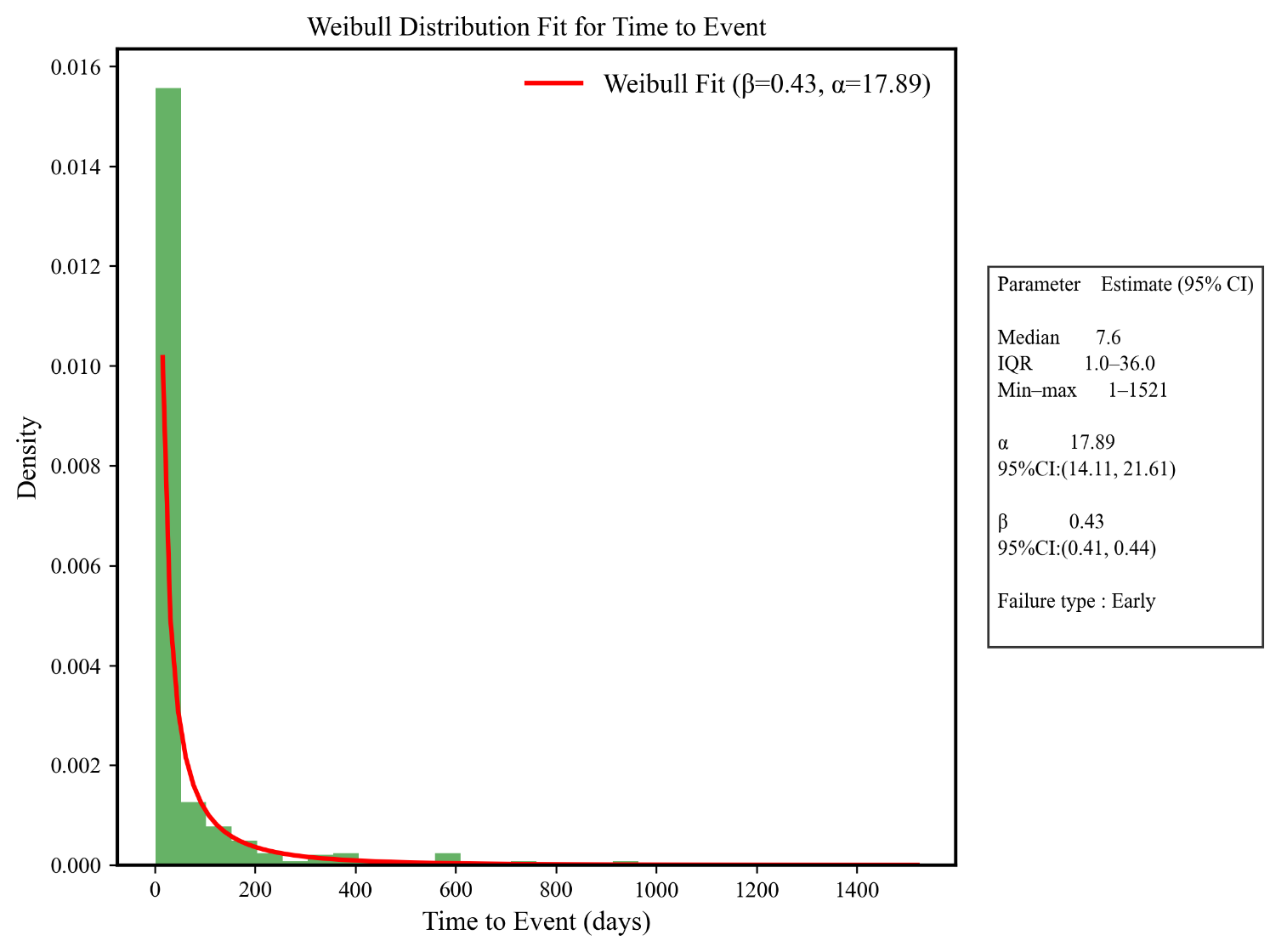


# HUMAN IMMUNOGLOBULIN G


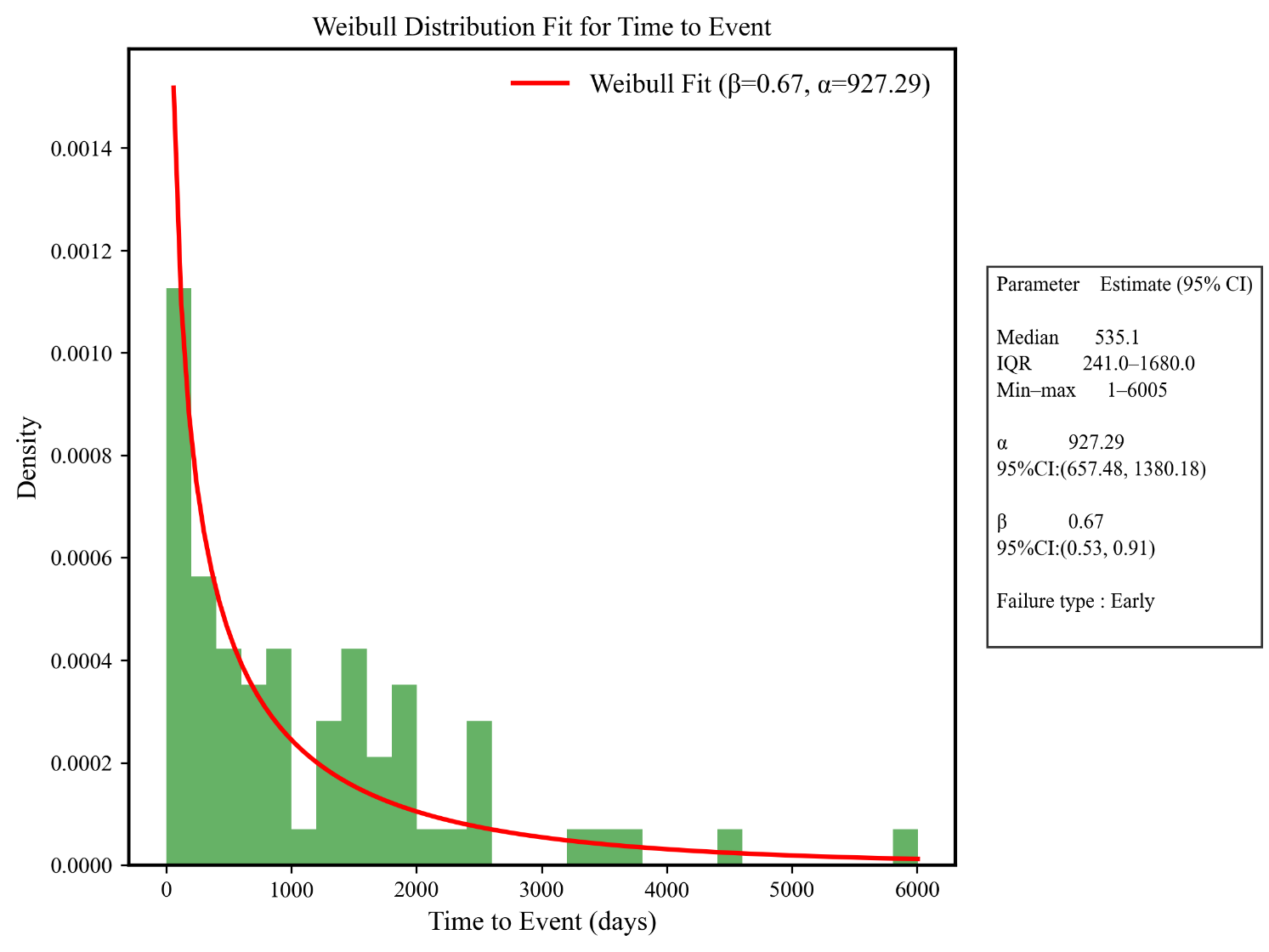


# INSULIN DEGLUDEC


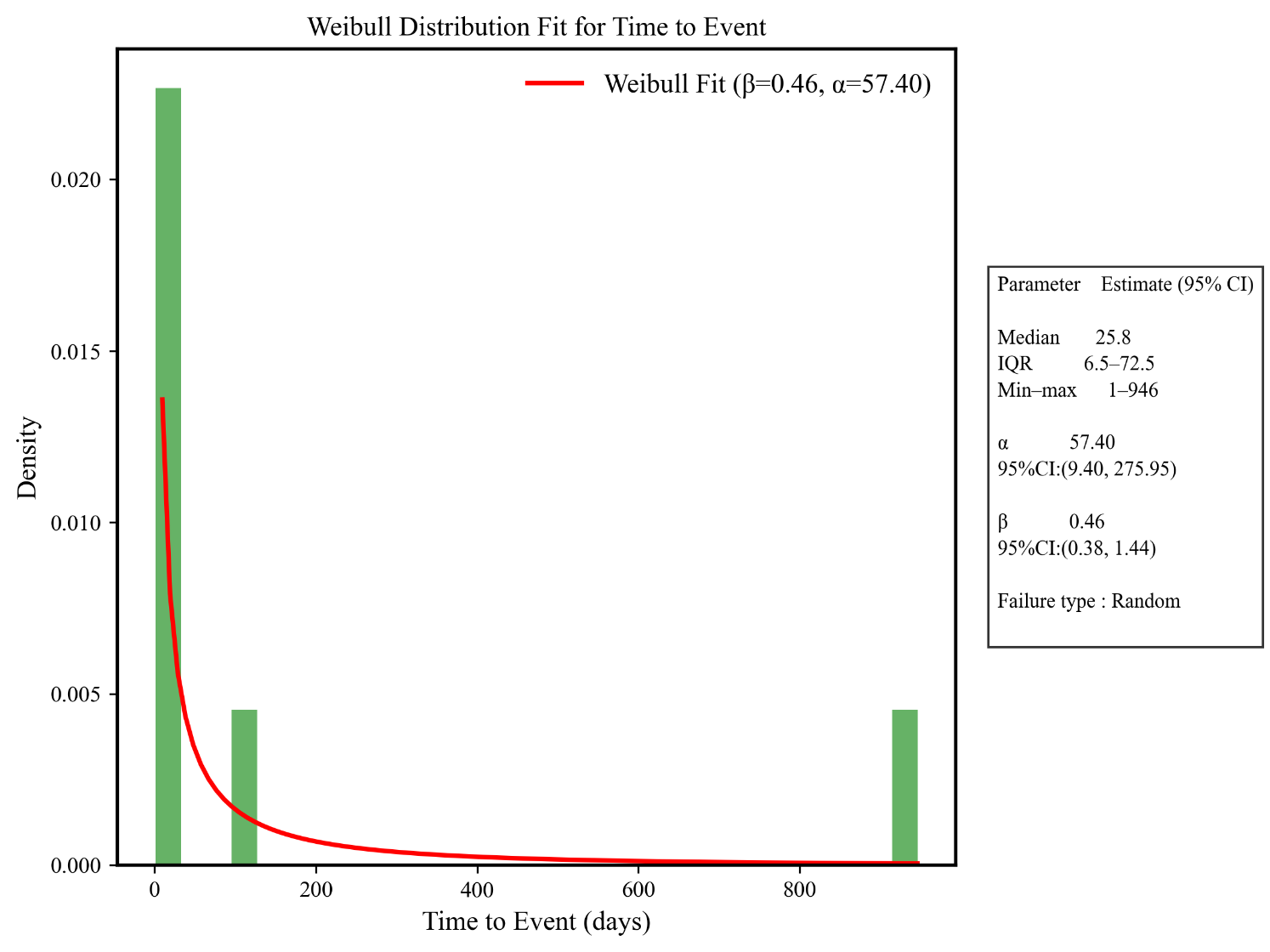


# INSULIN


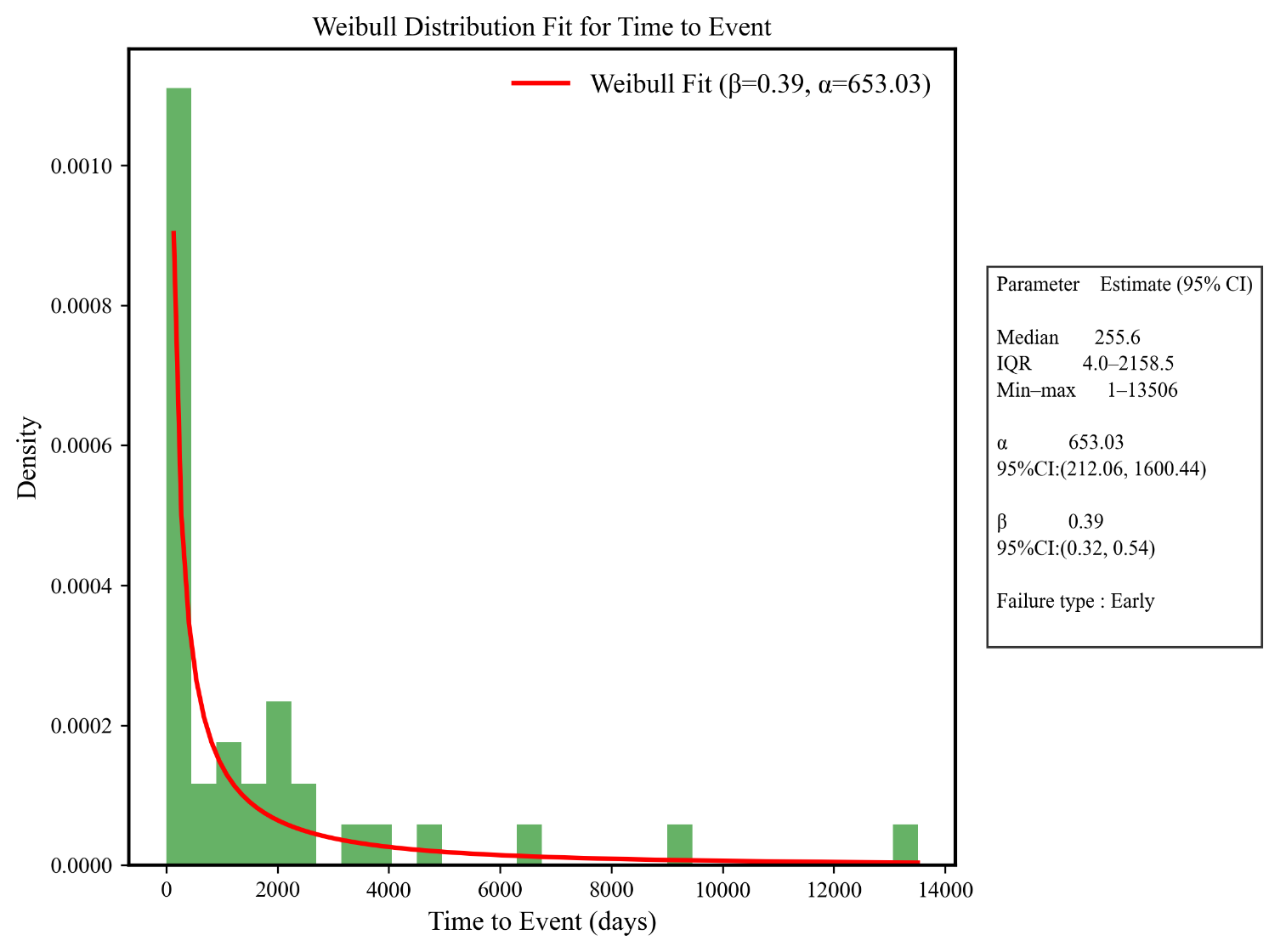


# LIRAGLUTIDE


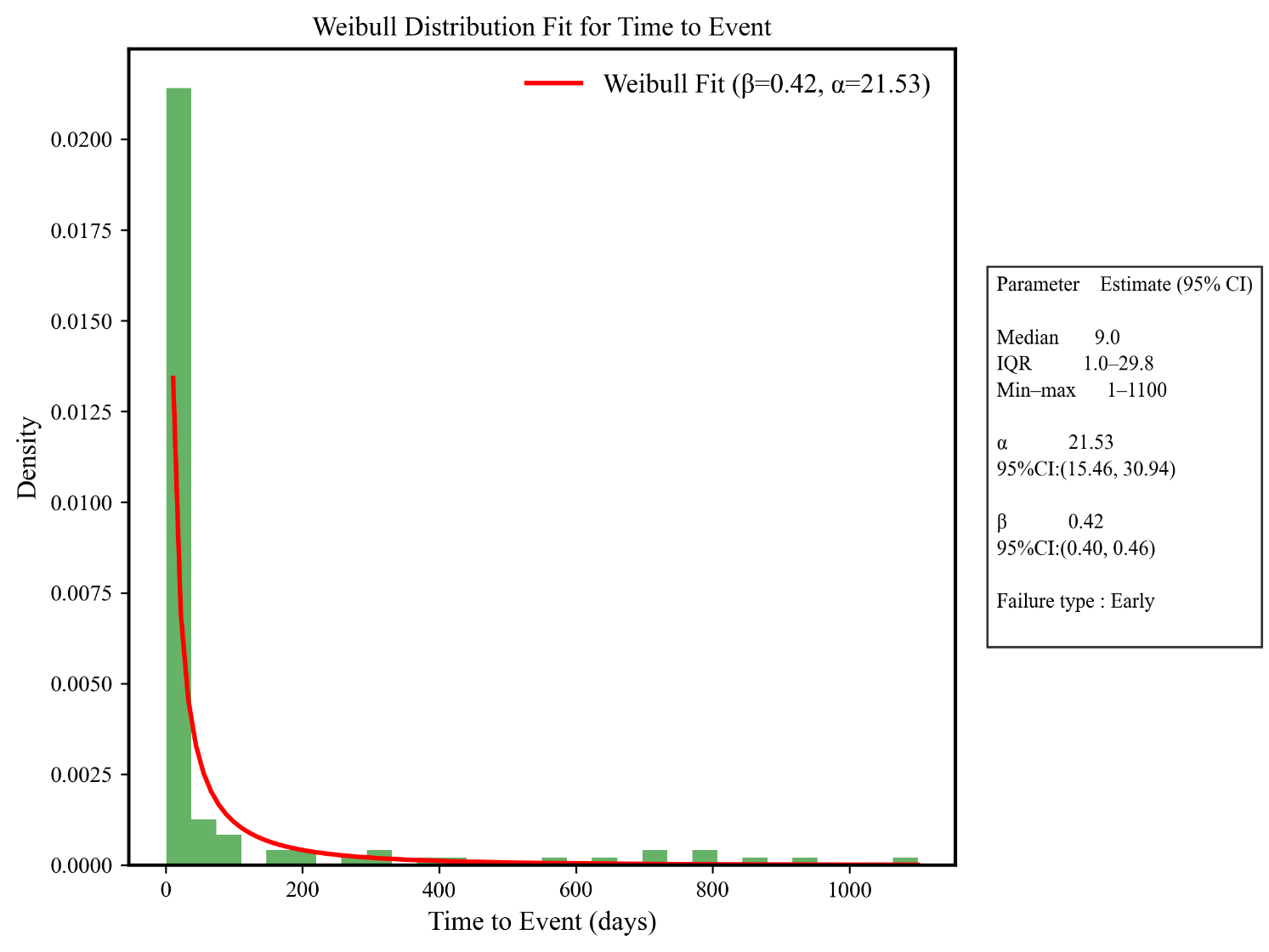


# METFORMIN


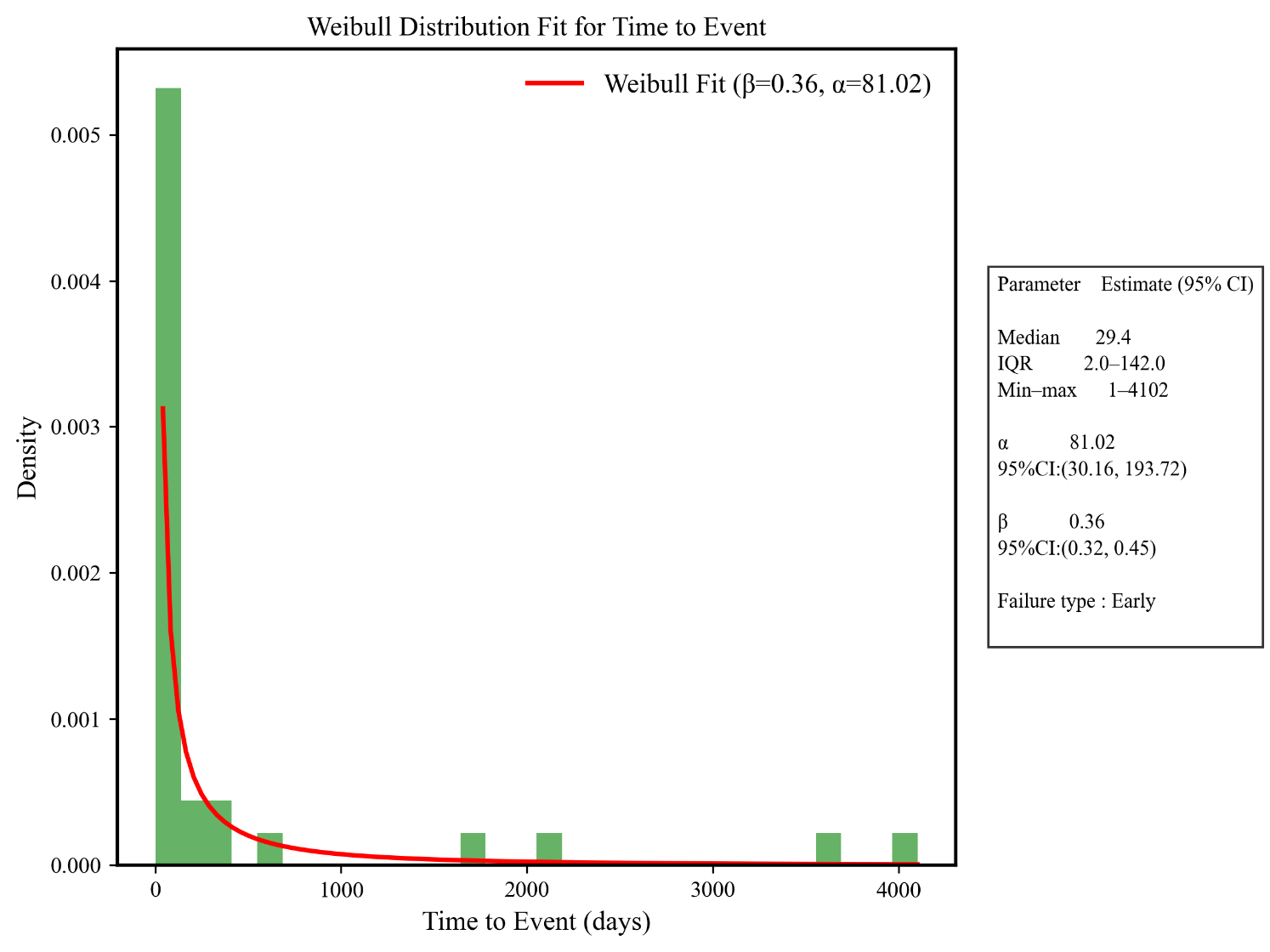


# METOCLOPRAMIDE HYDROCHLORIDE


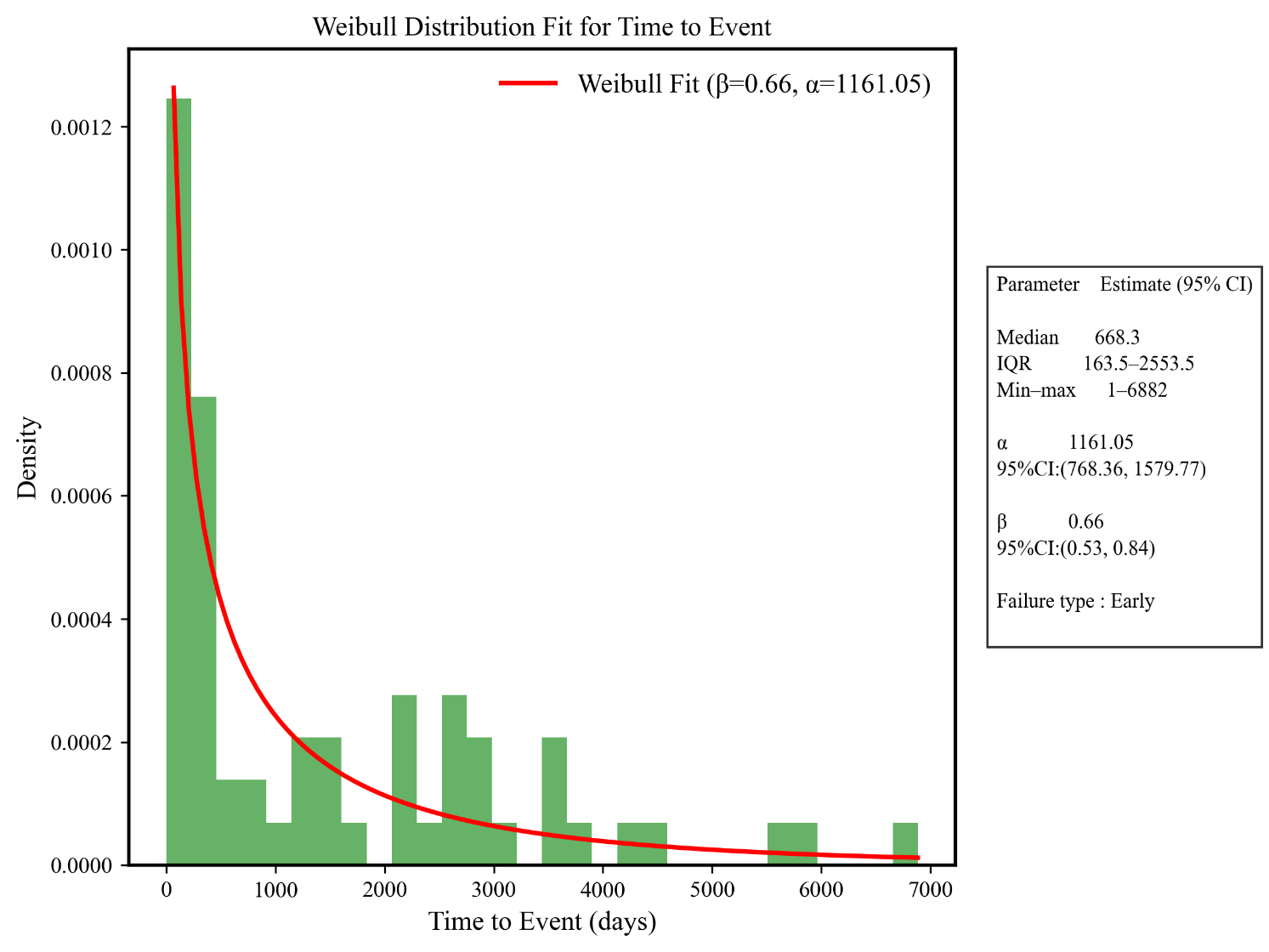


# OLMESARTAN MEDOXOMIL


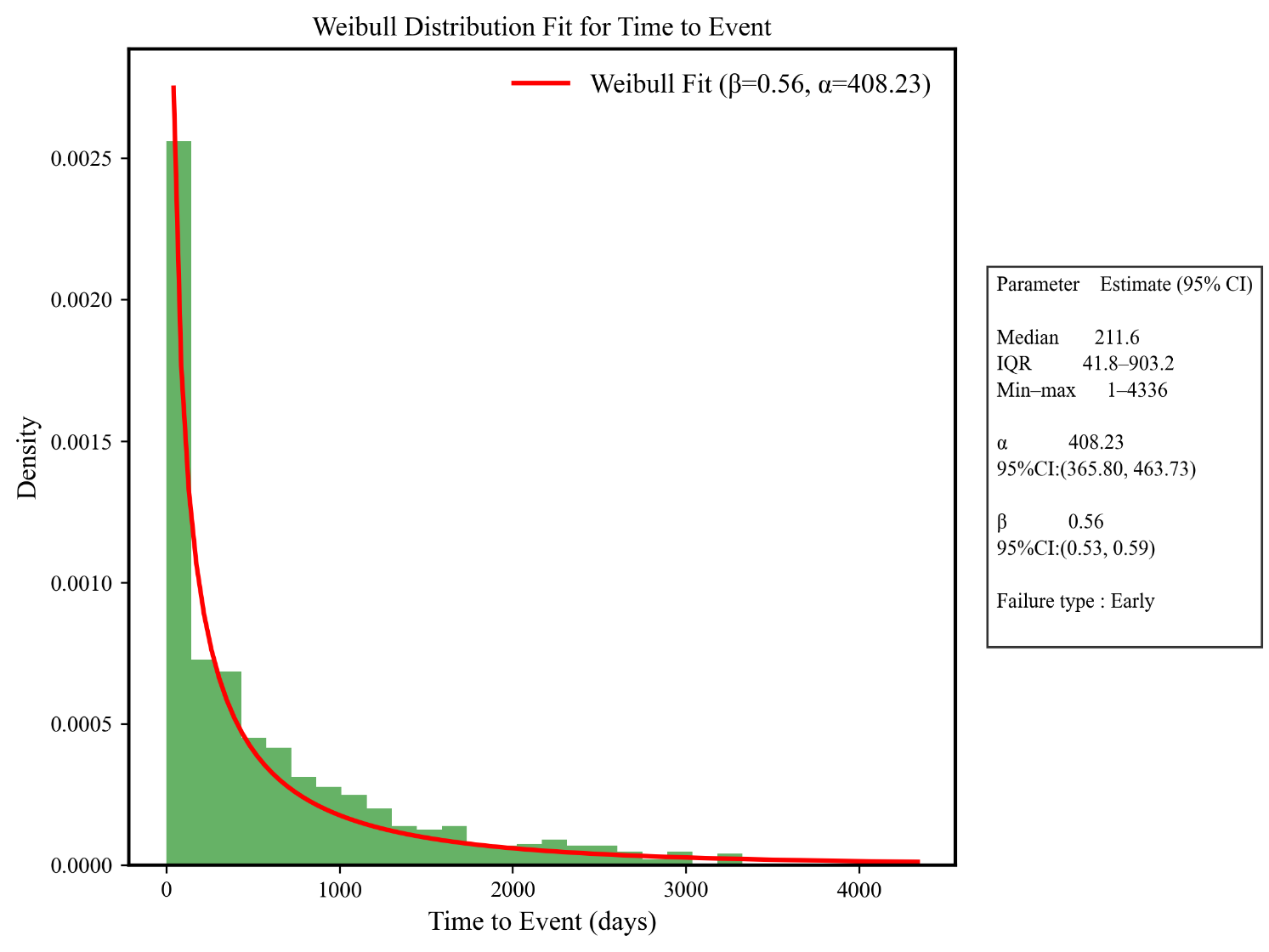


# PAMIDRONATE DISODIUM


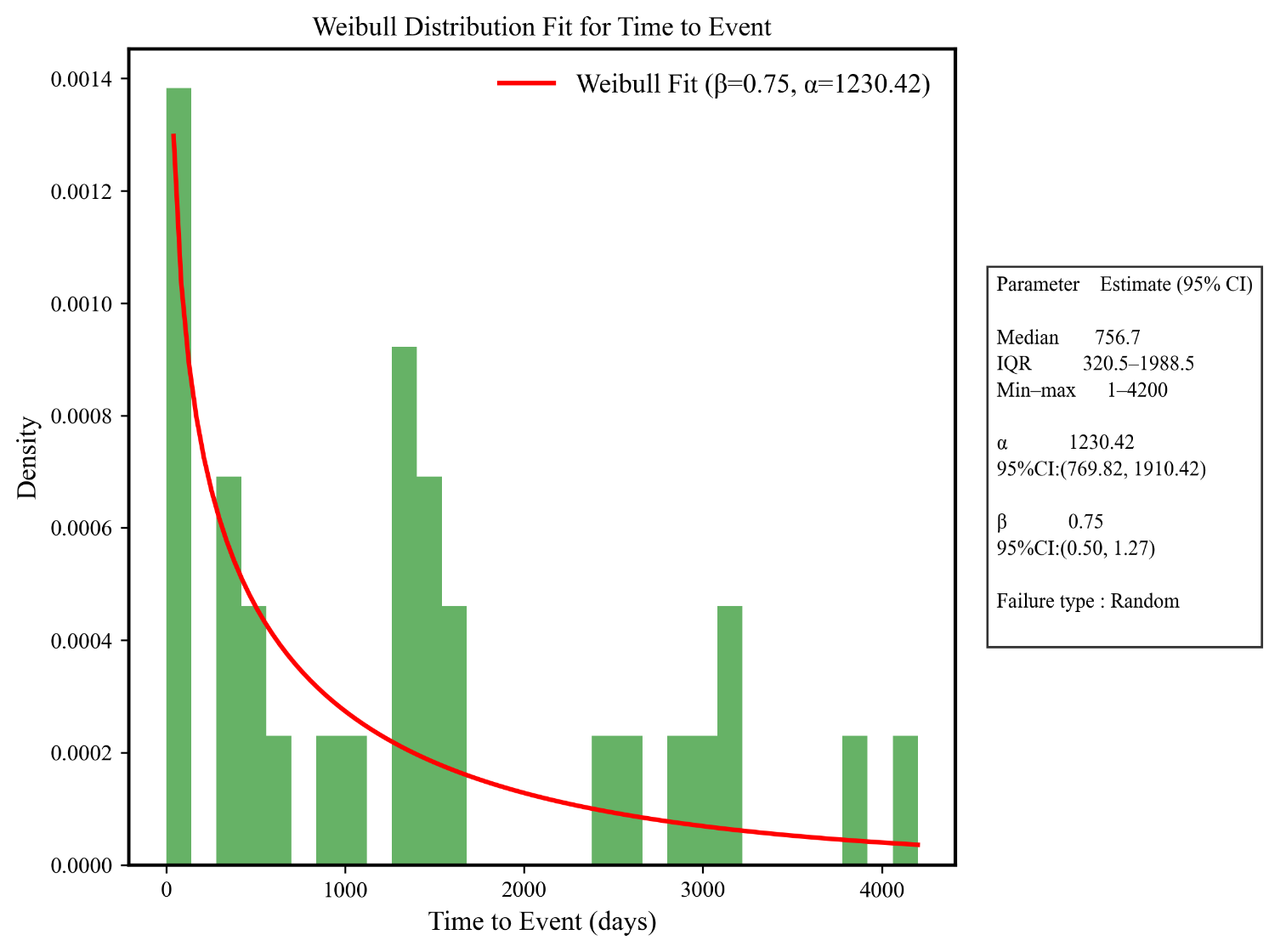


# PANCRELIPASE


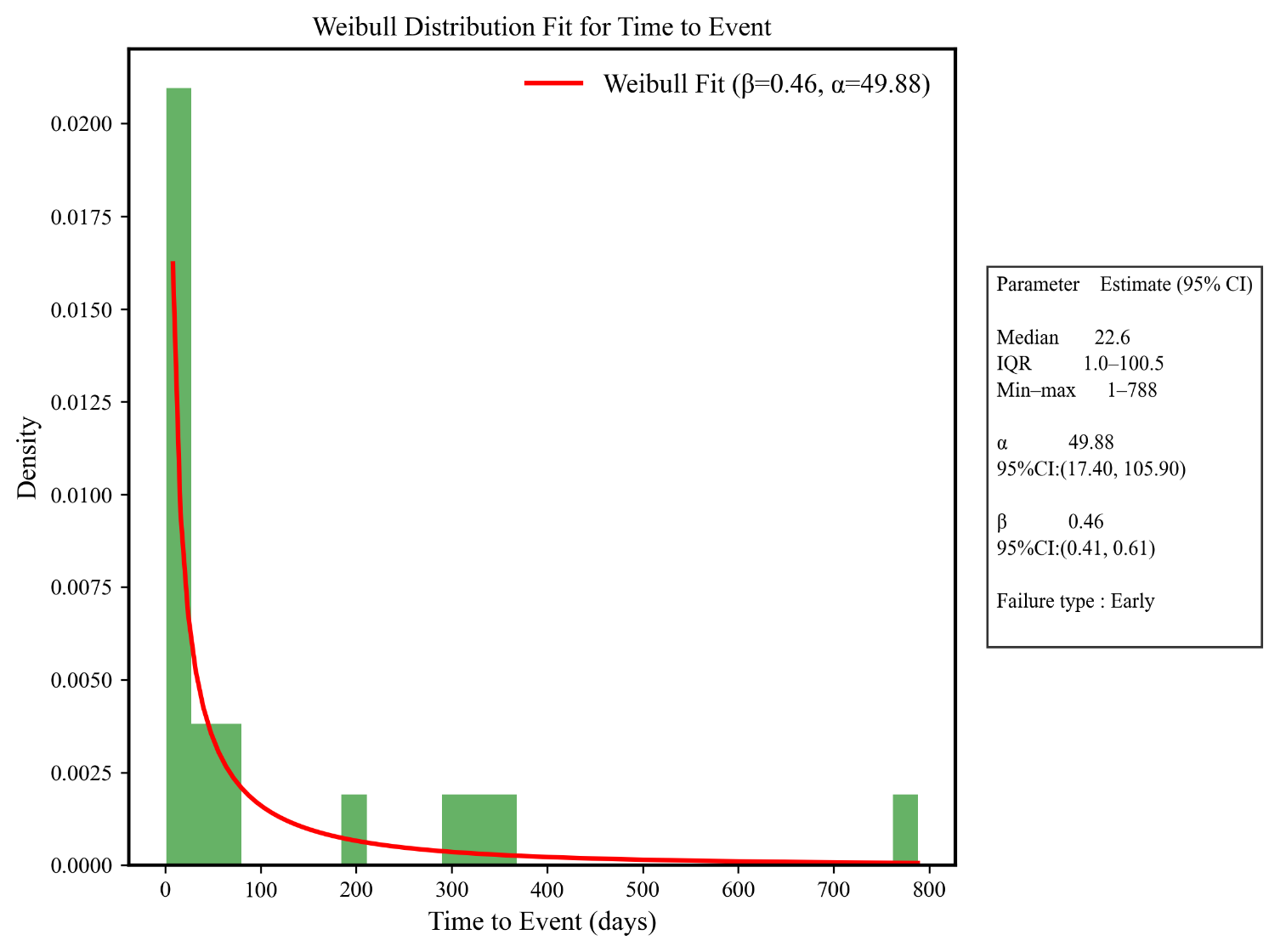


# SEMAGLUTIDE


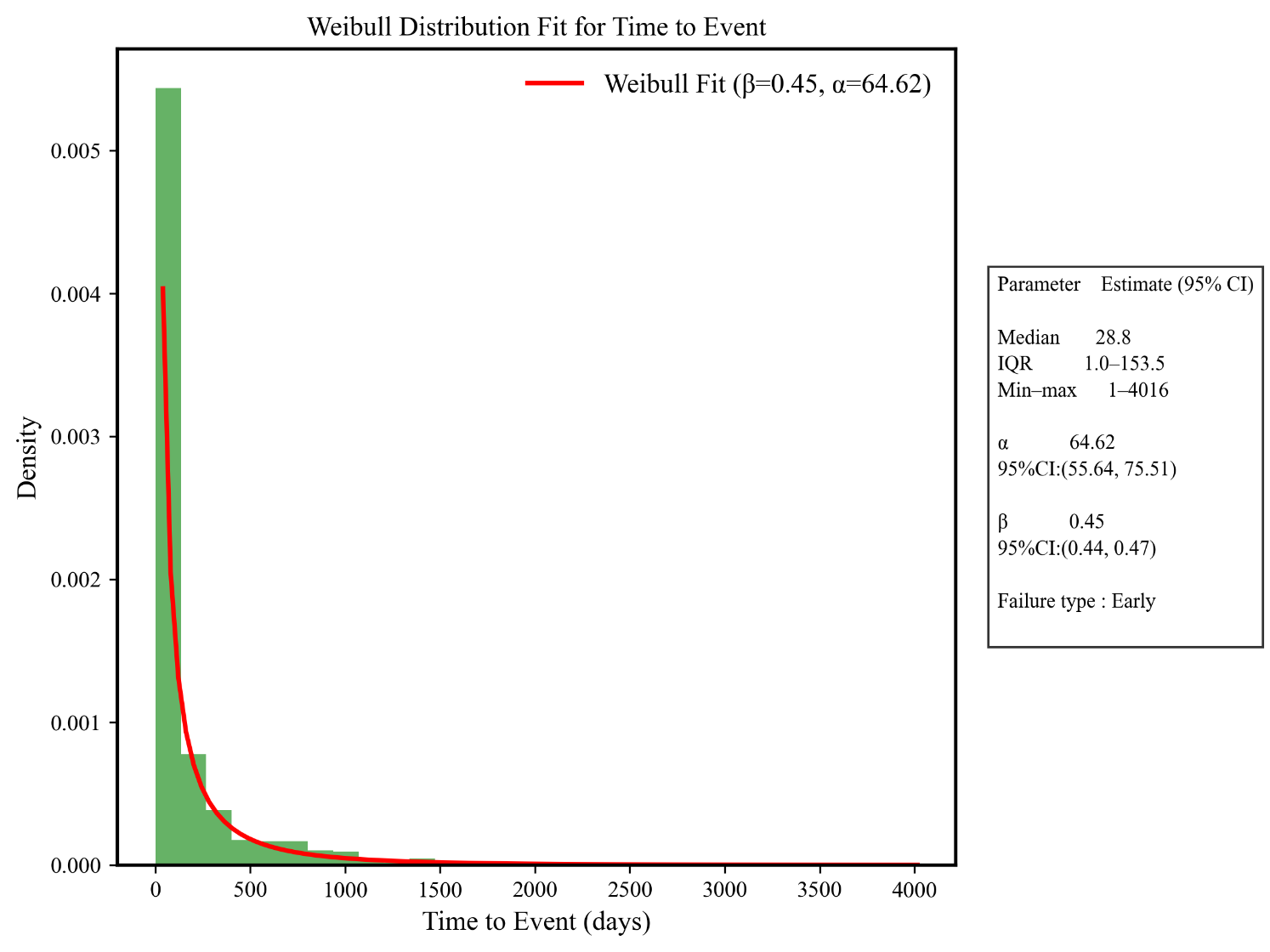


# SITAGLIPTIN PHOSPHATE


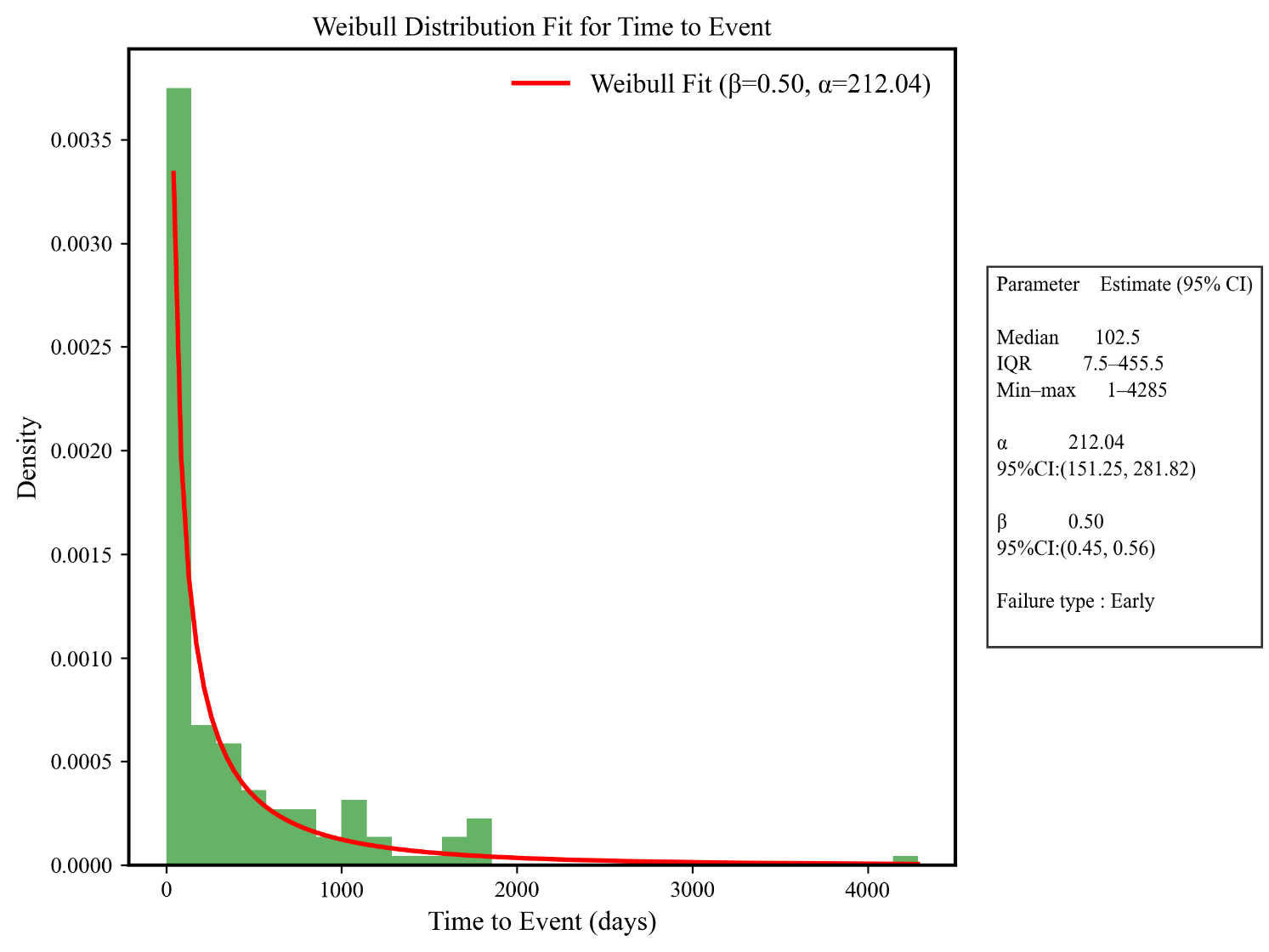


# SODIUM OXYBATE


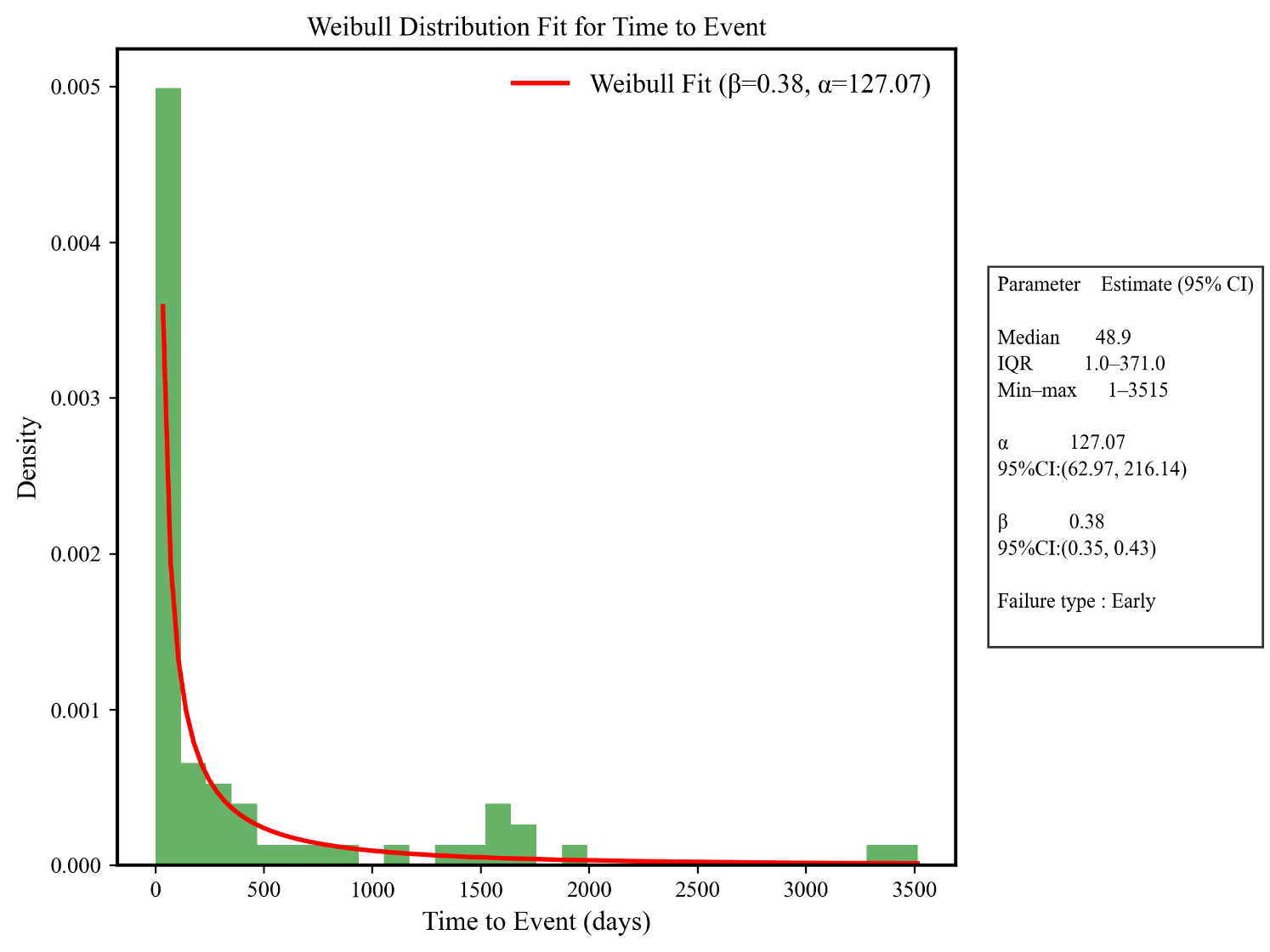


# TEDUGLUTIDE


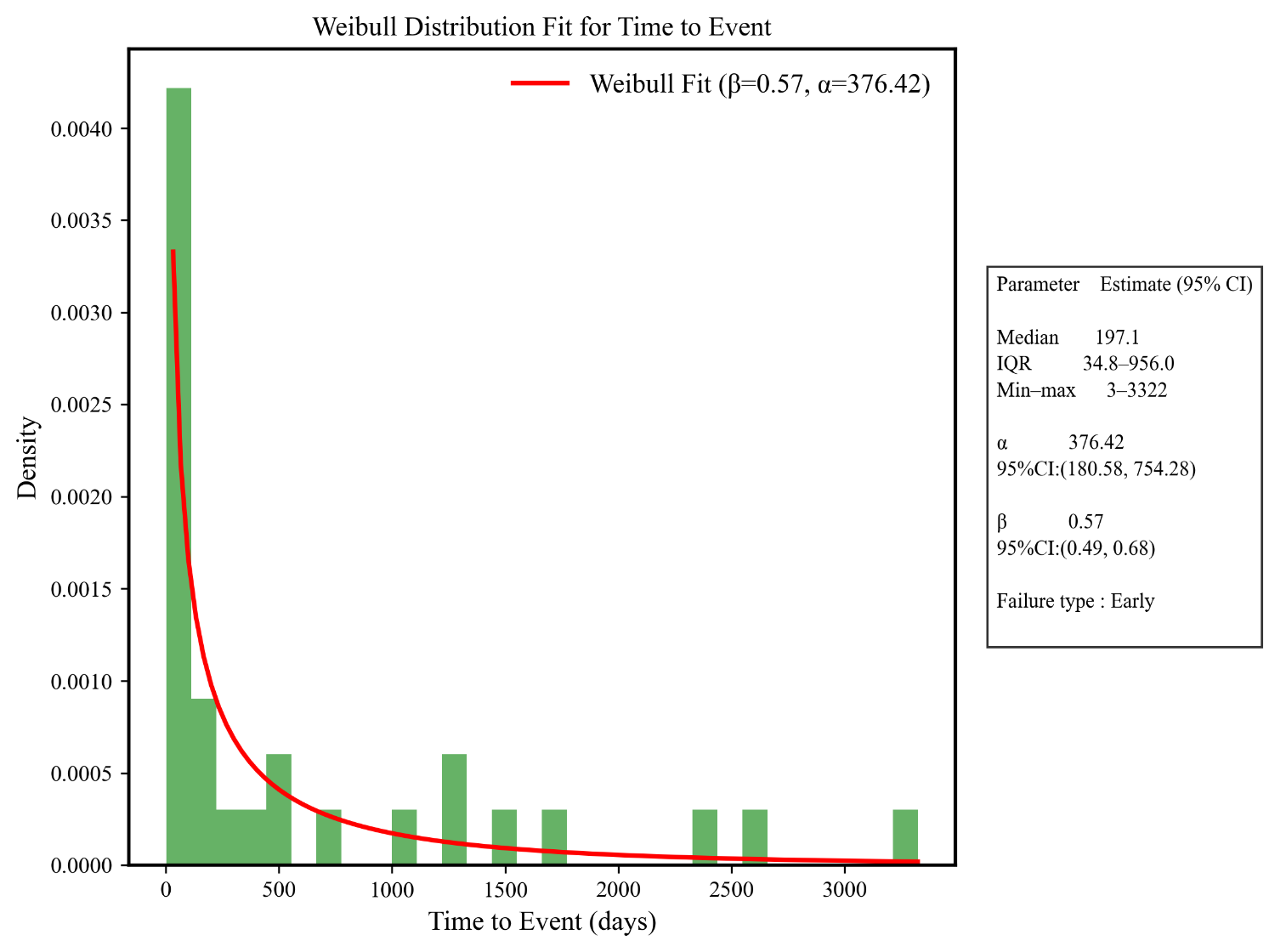


# TROFINETIDE


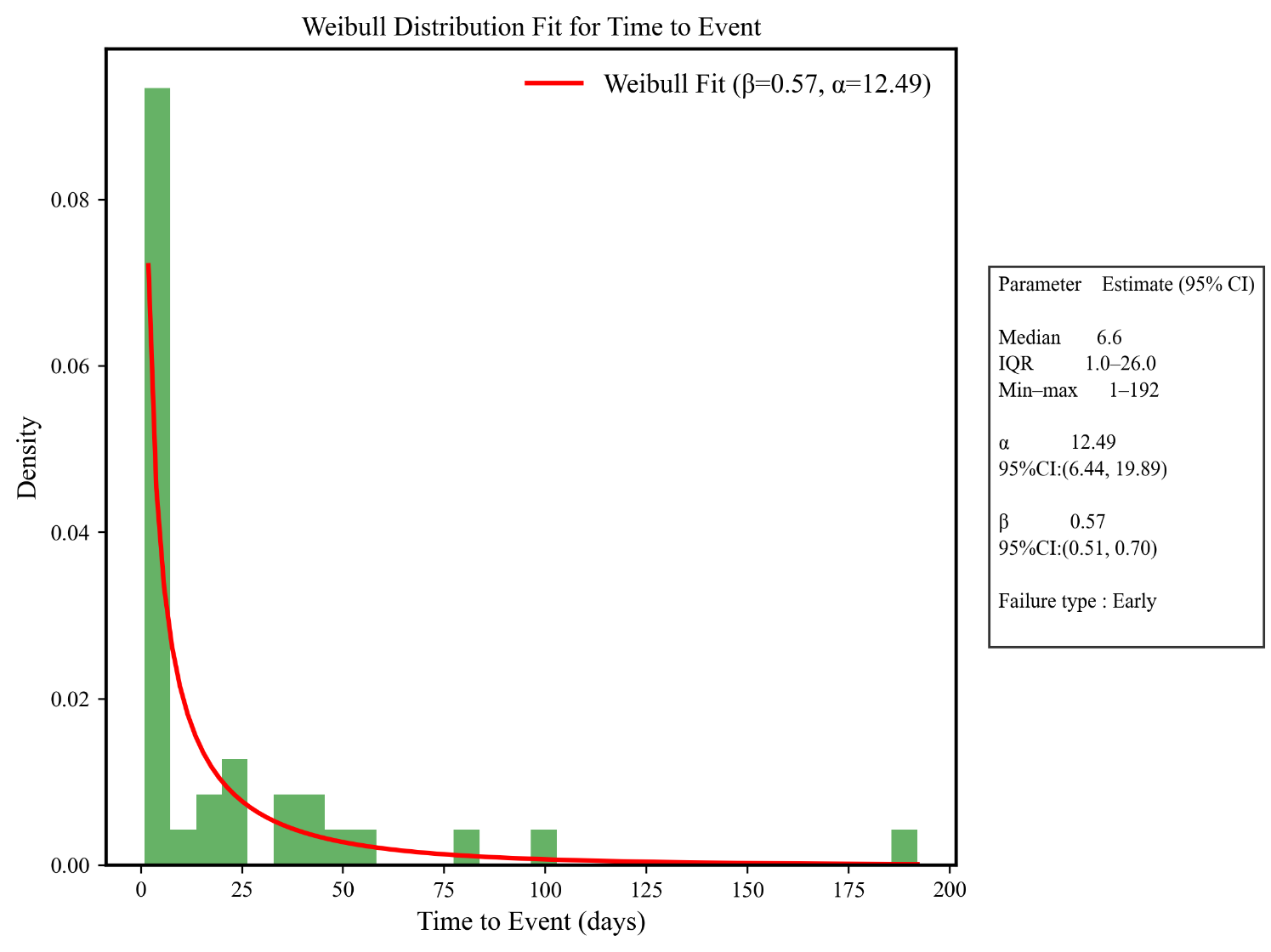

Supplement: S4 File — Weibull distribution fit for time-to-event data. The histogram (green bars) represents the observed probability density of time to event, with the red curve indicating the fitted Weibull distribution; Abbreviations: IQR, interquartile range; CI, confidence interval; Median, median time to event; β, Weibull shape parameter; α, Weibull scale parameter (days); Failure Type: Early, Failure risk decreases over time, with high incidence of early events, the 95% confidence interval of the shape parameter (β) does not contain 1, and β < 1; Random, The failure risk is constant, while events occur randomly, The 95% confidence interval of the shape parameter (β) contains 1. (DOCX) [file pone.0351731.s004.docx]
