# Supplementary material for: Drug-induced gastric motility disorders: A disproportionality analysis from the FAERS and CVARD databases
Source: PLoS One. 2026 Jun 12;21(6):e0351731. doi: 10.1371/journal.pone.0351731 (PMC13262828; doi:10.1371/journal.pone.0351731)
Supplement: Attachment — Submitted filename: Response_to_Reviewers.docx [file pone.0351731.s006.docx]

Response to Reviewers

Dear Academic Editor and Reviewers,

We sincerely thank the Academic Editor and both reviewers for their constructive and insightful comments on our manuscript entitled “Drug-Induced Gastric Motility Disorders: A Disproportionality Analysis from the FAERS and CVARD Databases.” We appreciate the positive assessment that the study is technically sound and that the data support the conclusions. We have carefully revised the manuscript according to the reviewers’ suggestions, with particular attention to improving clarity, standardizing the presentation of results, strengthening the Methods section, clarifying the interpretation of ROR and other disproportionality metrics, improving figure legends, and expanding the discussion of limitations related to external validation.

## Response to Reviewer #1

Comment 1:

*The article is very clear and the methodology is well described. The results are interesting, considering the widespread use of GLP-1 antagonists today and also in relation to insulin. It needs minor revisions, mainly in the writing. There are some typos that should be corrected, for example on line 98 (Mthod).*

Response:

We thank the reviewer for the positive assessment of our study and methodology. We corrected the typographical error in the section heading from “Mthod” to “Methods.” We also carefully reviewed the manuscript and corrected typographical and formatting errors throughout the revised version.

Location in revised manuscript:

Methods section heading, line 98.

Comment 2:

*It is also necessary to standardize the writing of the results, for example on lines 319 and 320.*

Response:

Thank you for this suggestion. We standardized the presentation of the Results section. Specifically, drug names were revised to avoid unnecessary capitalization, ROR values were supplemented with 95% confidence intervals where appropriate, and time-to-onset findings were reported using median values with interquartile ranges. We also standardized the presentation of the Weibull time-to-onset analysis, including the use of scale parameter α and shape parameter β.

Location in revised manuscript:

Results, Drugs associated with delayed gastric emptying and reflux, lines 171–191; Results, Heatmap of Reporting Odds Ratios (RORs), lines 212–241; Results, Weibull Time-to-Onset Analysis of Signal-Positive Drugs, lines 296–357; Table 4, around line 302.

## Response to Reviewer #2

Comment 1:

*Line 69–79 – What’s the reference group for the odds ratios in the analysis?*

Response:

Thank you for pointing this out. We clarified the reference framework in the Statistical analysis section. Specifically, we now state that disproportionality analysis was performed using a standard 2 × 2 contingency table, in which the reference framework consisted of reports involving the target drug with other adverse events and reports involving other drugs with and without the adverse event of interest.

Location in revised manuscript:

Methods, Statistical analysis subsection, lines 157–160.

Comment 2:

*Line 98 – Please correct the spelling error for the heading.*

Response:

Thank you for pointing this out. We corrected the heading to “Methods.”

Location in revised manuscript: Methods section heading, line 98.

Comment 3:

*Line 103 – Months or years would best describe the duration than the quarters.*

Response:

Thank you for this suggestion. We revised the description of the FAERS study period to include both the quarter-based range and the approximate duration in years. The revised manuscript now describes the dataset as covering the period from the first quarter of 2004 to the third quarter of 2025, approximately 21.75 years.

Location in revised manuscript:

Methods, Data sources and data cleaning subsection, lines 124–125.

Comment 4:

*Line 114 – Remove the upper case where not required.*

Response:

Thank you for pointing this out. This comment referred to the original Data Cleaning and Standardization subsection. We have rewritten this subsection and removed unnecessary capitalization where appropriate. The revised text now describes the cleaning and harmonization process in a clearer and more consistent manner, including SQL-based integration across FAERS quarters, harmonization according to the third-quarter 2025 documentation, MedDRA PT standardization, and removal of duplicate or unrecognizable records.

Location in revised manuscript:

Methods, Data sources and data cleaning subsection, lines 135–145.

Comment 5:

*Line 114–115 – The sentence is not comprehensible. Try to restructure it. Line 116–117 – same as above.*

Response:

Thank you for this comment. We rewrote the data cleaning subsection to improve clarity and readability. The revised text now describes the harmonization of FAERS data across quarters, the use of the third quarter of 2025 documentation as the harmonization standard, SQL-based processing, MedDRA PT standardization, and the removal of duplicate or unrecognizable records in a clearer sequence.

Location in revised manuscript:

Methods, Data sources and data cleaning subsection, lines 135–145.

Comment 6:

*Line 132 – It’s important to state the specific R package that was used in the analysis.*

Response:

We added the specific R packages used in the analysis. The revised Statistical analysis section states that data processing and analysis were performed using Microsoft Excel and R 4.4.2, with data.table and tidyverse for data management and processing, ggplot2 for visualization, and flexsurv for Weibull time-to-onset analysis.

Location in revised manuscript:

Methods, Statistical analysis subsection, lines 152–154.

Comment 7:

*For logical flow, the study design subheading should come first before other sections such as definition of variables and statistical analysis.*

Response:

We agree with the reviewer. We moved the Study design subsection to the beginning of the Methods section, before Data sources and data cleaning, Definition of variables, and Statistical analysis. This revised order better reflects the overall workflow of the study.

Location in revised manuscript:

Methods, Study design subsection, lines 99–115.

Comment 8:

*Line 137–139 – Why have the authors only indicated the ROR and not the other metrics as well? Further, I suggest the evaluation criteria using all the metrics be briefly described here and not just in the supplementary table. Also note that the evaluation criteria for all the three metrics, and not just the ROR, uses the lower bound limit of the 95% CI*

Response:

Thank you for this important suggestion. We revised the Statistical analysis section to describe the evaluation criteria for all three disproportionality metrics in the main text. The revised manuscript now states that ROR positivity required the lower bound of the 95% confidence interval to exceed 1; PRR positivity required PRR ≥ 2, χ² ≥ 4, and at least 3 cases; and BCPNN positivity required IC025 to exceed 0. We also clarified that a drug–preferred term pair was defined as a risk signal only when it simultaneously met the prespecified criteria of all three algorithms.

In addition, we clarified the role of ROR in the Results presentation. Although formal signal detection was based on concordance across ROR, PRR, and BCPNN, ROR was used primarily as an intuitive measure to present the relative magnitude of already signal-positive drug–event pairs.

Location in revised manuscript:

Methods, Statistical analysis subsection, lines 154–168.

Comment 9:

*Line 161–167 – There is no need to use upper cases for the drug names. Please revise the section. It is also important to list the drug in descending order by the signal strength.*

Response:

We revised this section accordingly. Drug names are no longer presented in all-uppercase format. We also reordered the 20 signal-positive drugs in descending order according to the highest ROR observed across all signal-positive preferred terms for each drug. This rule is explicitly stated in the revised Results section.

Location in revised manuscript:

Results, Drugs associated with delayed gastric emptying and reflux subsection, lines 186–191.

Comment 10:

*Line 170 and other sections with similar results – Include the central value, mean or median, with the spread for the onset of action.*

Response:

Thank you for pointing this out. We revised the time-to-onset results to report central values with measures of spread. Specifically, TTO is now presented primarily as median and interquartile range. We also revised the Weibull time-to-onset section and related Discussion passages to consistently report median TTO with IQR where appropriate.

Location in revised manuscript:

Results, Weibull Time-to-Onset Analysis of Signal-Positive Drugs, lines 296–357; Table 4, around line 302; Discussion, GLP-1 Receptor Agonists subsection, lines 381–390; Discussion, Other Therapeutic Classes subsection, lines 424–455.

Comment 11:

*Line 189 – Rephrase or delete the subheading.*

Response:

Thank you for this suggestion. We rephrased the subheading to make it more informative and less repetitive of the figure type. The previous subheading was revised to “Signal Distribution Across Drug–Event Pairs,” which better reflects the purpose of this section, namely to summarize the pattern and heterogeneity of ROR signals across the 20 signal-positive drugs and seven gastrointestinal preferred terms.

Location in revised manuscript:

Results, Signal Distribution Across Drug–Event Pairs subsection, lines 212–241.

Comment 12:

*Line 200, 201, 205 and all other sections with similar results – Include the 95% CI for the ROR.*

Response:

We revised the Results section to include 95% confidence intervals for ROR values where key signals are described. For example, the revised heatmap interpretation includes ROR values with 95% CIs for semaglutide, dulaglutide, liraglutide, exenatide, esomeprazole, trofinetide, olmesartan medoxomil, insulin degludec, and metoclopramide hydrochloride.

Location in revised manuscript:

Results, Signal Distribution Across Drug–Event Pairs subsection, lines 225–242; Results, Disproportionality Analysis of the CVARD Database for Validation Purposes, lines 255–280.

Comment 13:

*Line 213–220 – This section needs to be moved to the Methods section.*

Response:

We moved the relevant content describing the validation strategy and differential positivity criteria between FAERS and CVARD to the Study design subsection of the Methods section. The Results section now focuses on the validation findings rather than methodological justification.

Location in revised manuscript:

Methods, Study design subsection, lines 107–115.

Comment 14:

*The failure to validate other signals due to use of smaller Canadian database has not been well discussed in the section. Do you suggest another better database that can be used for further validation?*

Response:

Thank you for this important comment. We expanded the Limitations and Strengths section to discuss this issue more fully. We now clarify that failure to validate certain FAERS signals in CVARD should be interpreted cautiously rather than as evidence against the original associations. We explain that the smaller size of CVARD may limit statistical power for rare drug–event pairs and may result in wider confidence intervals. We also discuss differences in reporting practices, drug utilization patterns, market availability, and case accrual across countries as possible explanations for reduced cross-database concordance.

In addition, we added a Future Directions paragraph recommending further validation in additional pharmacovigilance resources. Specifically, we mention EudraVigilance as a broader external validation platform and JADER as a complementary resource for evaluating consistency in an East Asian reporting population.

Location in revised manuscript:

Discussion, Limitations and Strengths subsection, lines 551–561; Discussion, Future Directions subsection, lines 586–593.

Comment 15:

*Figure 2 – Confirm if some RORs have very narrow 95% CI or they are missing on the plot.*

Response:

We confirmed that the 95% confidence intervals were plotted for all drug–event pairs in Figure 2. For some pairs with relatively large case counts, the 95% CIs were extremely narrow and may visually overlap with the point estimates. In addition, for some pairs with very large ROR values or wide confidence intervals, the estimates extend beyond the plotted x-axis range and are truncated at the plot boundary for visualization. We revised Figure 2 by reducing the point size, using open circles, and slightly increasing the line width of the confidence intervals. We also added an explanatory note to the Figure 2 legend.

Location in revised manuscript:

Figure 2; Fig 2 legend, lines 176–185.

Comment 16:

*Figure 3 – Provide a more detailed description in the legend to help interpretation of the figure. For instance, what is the threshold for a signal on the log scale?*

Response:

Thank you for this comment. We reviewed the figure and clarified that Figure 3 presents raw ROR values, not log-transformed values. We therefore revised the figure and legend to avoid misleading reference to a log scale. The revised Figure 3 legend states that cell colors and numeric annotations indicate the corresponding raw ROR values. It also clarifies that blank cells indicate that no corresponding drug–PT entry was retained in the input dataset for that drug, rather than a separate color-based signal threshold. We further clarified that formal signal positivity was defined according to the prespecified concordant criteria described in the Methods section, rather than by a heatmap color threshold.

Location in revised manuscript:

Figure 3; Fig 3 legend, lines 217–224.

Comment 17:

*Revise to ensure logical flow of sections and information across the manuscript.*

Response:

We revised the manuscript structure to improve logical flow. The Study design subsection now appears at the beginning of the Methods section. The validation criteria were moved from the Results section to the Methods section. We also reorganized the Results and Discussion sections to reduce fragmentation and improve continuity.

Location in revised manuscript:

Methods, Study design subsection, lines 99–115; Methods, Statistical analysis subsection, lines 152–168; Results, Weibull Time-to-Onset Analysis of Signal-Positive Drugs subsection, lines 296–357; Discussion, Clinical Implications and Risk Mitigation Strategies subsection, lines 493–530.

Comment 18:

*Some subheadings are not necessary. Maintain only those that are important.*

Response:

Thank you for this comment. We removed several non-essential subheadings, particularly within the Weibull time-to-onset analysis section. We retained only subheadings that provide important structural guidance for readers.

Location in revised manuscript:

Results, Weibull Time-to-Onset Analysis of Signal-Positive Drugs subsection, especially lines 311–356; Discussion, Clinical Implications and Risk Mitigation Strategies subsection, lines 493–530.

Comment 19:

*There are too many short paragraphs. Where necessary, combine related information into one paragraph.*

Response:

We combined related short paragraphs throughout the manuscript where appropriate. For example, the baseline characteristics section, portions of the Weibull time-to-onset analysis, and selected Discussion subsections were revised to improve paragraph flow and reduce fragmentation.

Location in revised manuscript:

Results, Baseline characteristics of delayed gastric emptying and reflux subsection, lines 193–206; Results, Weibull Time-to-Onset Analysis of Signal-Positive Drugs subsection, lines 311–356; Discussion, GLP-1 Receptor Agonists subsection, lines 370–398; Discussion, Clinical Implications and Risk Mitigation Strategies subsection, lines 493–530.

Comment 20:

*You stated that signal detection was based on all the metrics, ROR, PRR and IC025, but in the results, the ROR is the most outstanding metric. Please make sure you highlight this in the methods and discussion sections.*

Response:

Thank you for this important clarification. We highlighted this point in both the Methods and Discussion sections. In the Methods section, we state that formal signal detection was based on concordance across ROR, PRR, and BCPNN, while ROR was used in the Results as an intuitive measure to present the relative magnitude of already signal-positive drug–event pairs. In the Discussion section, we added a sentence clarifying that, for clarity and brevity, discussion of signal strength is presented primarily using ROR values, whereas formal signal detection was based on concordance across ROR, PRR, and BCPNN.

Location in revised manuscript:

Methods, Statistical analysis subsection, lines 166–168; Discussion, Principal Findings and Pharmacological Implications subsection, lines 366–368.

We thank the Academic Editor and reviewers again for their helpful comments, which have substantially improved the clarity, organization, and interpretability of the manuscript.

Sincerely,

Zhiheng Qian and co-author(s)
